# Nutrient legacies from elephant megacarcasses create coldspots of biodiversity and hotspots of ecosystem processes

**DOI:** 10.1073/pnas.2605332123

**Published:** 2026-10-12

**Authors:** Courtney G. Reed, Nathan P. Lemoine, Michelle L. Budny, Vincent C. Clarke, Johan T. du Toit, Ryan Helcoski, Helga Knoetze, Jane M. Lucas, Joshua P. Schimel, Izak P. J. Smit, Tercia Strydom, Aimee G. Tallian, Dave I. Thompson, Deron E. Burkepile

**Affiliations:** ^a^ Marine Science Institute, University of California, Santa Barbara, CA 93106; ^b^ Department of Biological Sciences, Marquette University, Milwaukee, WI 53233; ^c^ Department of Zoology, Milwaukee Public Museum, Milwaukee, WI 53233; ^d^Unit for Environmental Sciences and Management, North West University, Potchefstroom 2520, South Africa; ^e^ Department of Wildland Resources and the Ecology Center, Utah State University, Logan, UT 84322; ^f^ Institute of Zoology, Zoological Society of London, London NW1 4RY, England, United Kingdom; ^g^The Expanded Freshwater and Terrestrial Environmental Observation Network, Kimberley 8301, South Africa; ^h^ Cary Institute of Ecosystem Studies, Millbrook, NY 12545; ^i^ Department of Ecology, Evolution, and Marine Biology, University of California, Santa Barbara, CA 93106; ^j^ Scientific Services, South African National Parks, George 6529, South Africa; ^k^ Sustainability Research Unit & CNRS-NMU International Research Laboratory, REHABS, Nelson Mandela University, George 6529, South Africa; ^l^ Department of Zoology and Entomology, University of Pretoria, Pretoria 0001, South Africa; ^m^ Scientific Services South African National Parks, Skukuza 1350, South Africa; ^n^ Soil, Crop, and Climate Sciences, University of the Free State, Bloemfontein 9301, South Africa; ^o^ Division of Land and Biodiversity, Norwegian Institute for Nature Research, Trondheim 7485, Norway; ^p^ South African Environmental Observation Network, Ndlovu Node, WITS Rural Campus, Acornhoek 1360, South Africa; ^q^ School of Animal, Plant and Environmental Sciences, University of the Witwatersrand, Johannesburg 2050, South Africa

**Keywords:** carrion, ecosystem function, zoogeochemistry, megafauna, nutrient cycling

## Abstract

Terrestrial megafauna (animals >1,000 kg) have substantial impacts on ecosystems while alive; yet, we know little about the impacts of these ecosystem engineers after they die. Like whale carcasses in the ocean, terrestrial megacarcasses may leave ecological legacies via the nutrient pulses they create on the landscape. Here, we investigated the impact of elephant megacarcasses on savanna ecosystems in Kruger National Park, South Africa. To determine how elephant carcasses affect below- and aboveground biodiversity and ecosystem processes, we collected soil and plant samples from 40 carcass sites (pairing “on carcass” and “off carcass” samples) ranging from 8 d to 7.7 y postmortem. We found that elephant carcasses released enormous pools of nutrients into savanna soils, increasing ammonium by 31×, nitrate by 7×, and phosphorus by 3× on average. These extreme ammonium concentrations were negatively correlated with the biodiversity of bacteria and plants, which were both suppressed at elephant carcass sites. Despite these decreases in biodiversity, ecosystem processes were strongly elevated at elephant carcass sites, with soil microbial respiration elevated for 1 to 2 y and herbivore grazing >3× higher for 7+ y postmortem. These results demonstrate that elephant megacarcasses generate coldspots of biodiversity but hotspots of ecosystem processes, unveiling a cryptic ecological function of megaherbivores that is extreme in both magnitude and duration relative to carcasses of smaller animals. As humans drive terrestrial megafauna toward extinction, we risk losing not only their ecosystem engineering abilities while they are alive but also the ecological legacies their carcasses create after death.

## Significance

Megaherbivores have transformative impacts on ecosystems during their lives; yet, we know little about their continued ecosystem engineering effects after they die. We investigated the legacy effects of elephant megacarcasses on savanna ecosystems and found that they release enormous pools of nutrients into soil, dramatically reducing the biodiversity of microbes and plants. Despite these reductions in biodiversity, elephant megacarcasses enhance ecosystem processes by stimulating soil microbial activity and facilitating herbivory on nutrient-rich plants. These impacts persist for 7+ y after elephants die and are extreme in magnitude and duration relative to the carcasses of smaller animals. Our findings demonstrate the role of megacarcasses in regulating nutrient cycling, biodiversity, and ecosystem processes.

Animals influence community dynamics and ecosystem function—shaping patterns of species diversity, determining transitions between ecosystem types, controlling the cycling of nutrients, and influencing fire regimes (1, 2). Most studies focus on the roles animals play in ecosystems while they are alive; yet animals can leave ecological legacies after they die. Across ecosystems, animal carcasses, ranging from cicadas (*Magicicada* spp.) to wildebeest (*Connochaetes taurinus*) to moose (*Alces alces*), can have lasting effects on ecological communities and processes, altering nutrient cycling and species diversity (3–5). Yet the effects of the carcasses of terrestrial megafauna (animals >1,000 kg) remain largely unexplored, despite their potential to release enormous nutrient pulses into soils (6) and influence ecological dynamics across multiple scales. In the ocean, megacarcasses of great whales (“whale falls”) deposit large pulses of nutrients to the ocean floor, creating hotspots of biodiversity and ecosystem processes that last decades (7, 8). Surprisingly, we know more about the legacies of whale falls in the deep sea than we do about terrestrial megacarcasses, despite the fact that our remaining megaherbivores (e.g., elephants and rhinoceros) are ecosystem engineers that, in life, transform habitats and influence ecosystem processes (9, 10).

The carcasses of terrestrial animals leave behind nutrient-rich legacies that shape both belowground and aboveground components of ecosystems. Belowground, smaller carcasses such as deer (*Odocoileus virginianus*) and rabbits (*Oryctolagus cuniculus*) can create nutrient hotspots that increase soil inorganic nitrogen (N) by up to 8× and phosphorus (P) by 2× (11, 12). These nutrient hotspots can increase soil microbial biomass, alter microbial community composition, and stimulate soil respiration and organic matter decomposition (5, 13, 14). Yet, nutrient hotspots and their impacts from smaller carcasses may be short lived. For example, nutrients in the soils below roe deer (*Capreolus capreolus*) carcasses did not differ from controls after ~2 y (15), and even carcasses of the largest non-megafauna such as bison (*Bos bison*), elk (*Cervus canadensis*), and moose create nutrient legacies lasting <5 y (5, 13, 16). Aboveground, nutrient inputs from carcasses such as bison and kangaroos (*Macropus giganteus*) alter plant community composition and diversity (16, 17), while moose carcasses facilitate tree recruitment at the expense of herbaceous plants (11). Increased soil N at vicuña (*Lama vicugna*) carcass sites also cause increased foliar N and thereby increased herbivory (18). Thus, the nutrients from terrestrial carcasses can influence a number of community dynamics and ecosystem processes.

Megacarcasses likely produce ecological legacies that are both more intense and longer-lasting than those of smaller animals. African savanna elephants (*Loxodonta africana*) are the largest terrestrial animals and are orders of magnitude larger than most other animals, with bodies up to 6,000 kg containing hundreds of kg of C, N, and P that are deposited into the savanna when they die. As such, soil N and P are strongly elevated at fresh elephant carcass sites (6). Yet, the long-term impacts of terrestrial megacarcasses on nutrient dynamics, biodiversity, and ecosystem processes are unknown, representing an important gap in our knowledge about the role of animal carcasses in terrestrial ecology. Understanding the ecological impacts of terrestrial megafauna both in life and after death is critical because these species are rapidly being lost due to human exploitation and habitat loss (19). If megacarcasses function as hotspots of nutrient input and ecosystem activity, then the loss of these animals represents a double jeopardy: Not only do we lose the living ecosystem engineers, but we also forfeit the prolonged ecological legacies created by their carcasses.

**Fig. 1.**
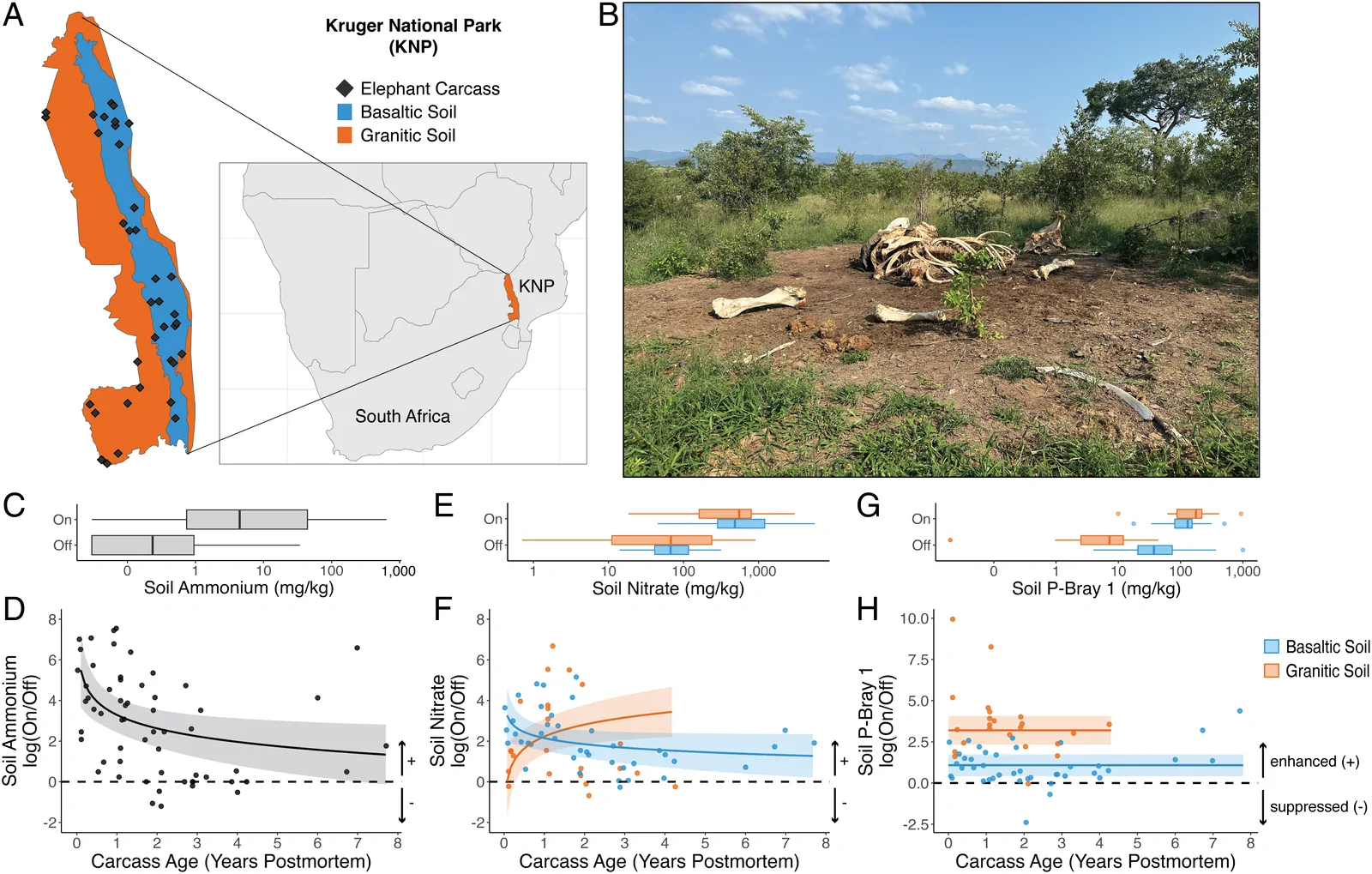
Impact of elephant carcasses on soil nutrient concentrations. (*A*) Samples were taken from 40 elephant carcass sites in Kruger National Park, South Africa. Carcasses ranged in age from 8 to 2,814 d (0 to 7.7 y) postmortem and were divided across granitic (N = 13) and basaltic (N = 27) soils. (*B*) Image of a 6 wk-old carcass on granitic soil, characterized by reduced vegetation and a persistent bonefield. Elephant carcasses had strong positive effects on soil (*C* and *D*) ammonium, (*E* and *F*) nitrate, and (*G* and *H*) plant-available phosphorus that persisted even at the oldest carcasses. Panels *C*, *E*, and *G* show the distribution of values for each soil nutrient on vs. off carcass sites in which boxes show the interquartile range, the middle line indicates the median, whiskers extend up to 1.5× the interquartile range, and points are shown for outliers that fall >1.5× outside the interquartile range. For panels *D*, *F*, and *H*, points represent on vs. off carcass log response ratios (LRRs) from individual carcass sites, where on carcass is the mean nutrient concentration for samples taken at 0.5 and 2.5 m distances and off carcass is the concentration for samples taken at 15 m. Points are offset to be visible when they would otherwise overlap. Points above the dashed horizontal line at 0 indicate positive effects of carcasses on the soil nutrient, while points below 0 indicate negative effects. For reference, an LRR of 2 means the value on the carcass is ~7× greater than off, while an LRR of 8 is ~3,000× greater. Lines show predictions calculated from the linear mixed effects model, which included: i) the log of carcass age, ii) soil type, and iii) a log of carcass age × soil type interaction as covariates. Only significant relationships are shown on the plots. Thus, panels *F* and *H* have different colors for granitic and basaltic soils as there were significant year postmortem × soil type interactions, while panel *D* has no color distinction for soil as there was only a significant effect of carcass age. Shading indicates the 95% CI.

Here, we investigated the ecological legacies of elephant carcasses on belowground and aboveground ecosystem dynamics in the Kruger National Park (KNP), South Africa. We collected data from 40 elephant carcass sites ranging from 8 to 2,814 d (0 to 7.7 y) after death across two different soil types (Fig. 1 *A* and *B*) to ask how elephant carcasses affect soil nutrient dynamics, below- and aboveground biodiversity, and ecosystem processes. Although previous research shows significant effects of carcasses both below- and aboveground, rarely do studies examine how these carcass legacies propagate across levels of ecological organization (20). We hypothesized that megacarcasses would release extreme amounts of nutrients that would: i) create sustained nutrient hotspots of N and P in soils, ii) restructure microbial and plant communities through nutrient-driven shifts in diversity, iii) increase rates of belowground microbial respiration, and iv) attract herbivores and increase herbivory in response to elevated foliar nutrients. We expected these integrated ecological legacies to persist for years after an elephant’s death.

## Results

### Megacarcasses Created Persistent Nutrient Hotspots in Soils.

Consistent with our expectations, soil nutrient concentrations were elevated at carcass sites (*SI Appendix*, Table S1). Soil ammonium and nitrate were higher on carcass sites vs. off carcass (mean ~31× greater for ammonium and ~7× higher for nitrate), indicated by positive intercepts and CI > 0 across the chronosequence, since an LRR > 0 indicates the nutrient was elevated on carcass sites relative to off carcass (lmm: Benjamini-Hochberg corrected *P*-value (*q*) *=* 0.004 for both). The effect of the carcass on soil ammonium decreased with carcass age (*q* = 0.012) but still remained positive on average (Fig. 1 *C* and *D*). Soil nitrate exhibited a significant carcass age × soil type interaction (*q* = 0.004; Fig. 1 *E* and *F*), although nitrate remained elevated at carcass sites of both soil types across the chronosequence. Elephant carcasses had a significant positive effect on soil δ^15^N (*q* = 0.004) that may have shown a slight decrease with carcass age (*q* = 0.052), showing that the N in the soils at the carcass sites was animal-derived (*SI Appendix*, Fig. S1).

The effect of elephant carcasses on soil P was also large and long-lasting (*SI Appendix*, Table S1). Soil plant-available P was on average ~3× greater on carcass sites relative to off carcass sites (lmm: *q* = 0.004; Fig. 1 *G* and *H*). This effect was greater in granitic soils than in basaltic soils (*q* = 0.004), and the effect remained strong across the 7.7-y time period (*q* = 0.945). Soil pH did not differ significantly on vs. off carcass sites (lmm: *q* = 0.078), but there was a significant carcass age × soil type interaction (*q* = 0.004) in which pH for basaltic soils appeared to decrease slightly and remain suppressed over time, while pH for granitic soils showed an initial increase before rapidly decreasing over the first year postmortem (*SI Appendix*, Fig. S2 and Table S1).

### Megacarcasses Suppressed Soil Microbial Diversity but Enhanced Soil Respiration.

**Fig. 2.**
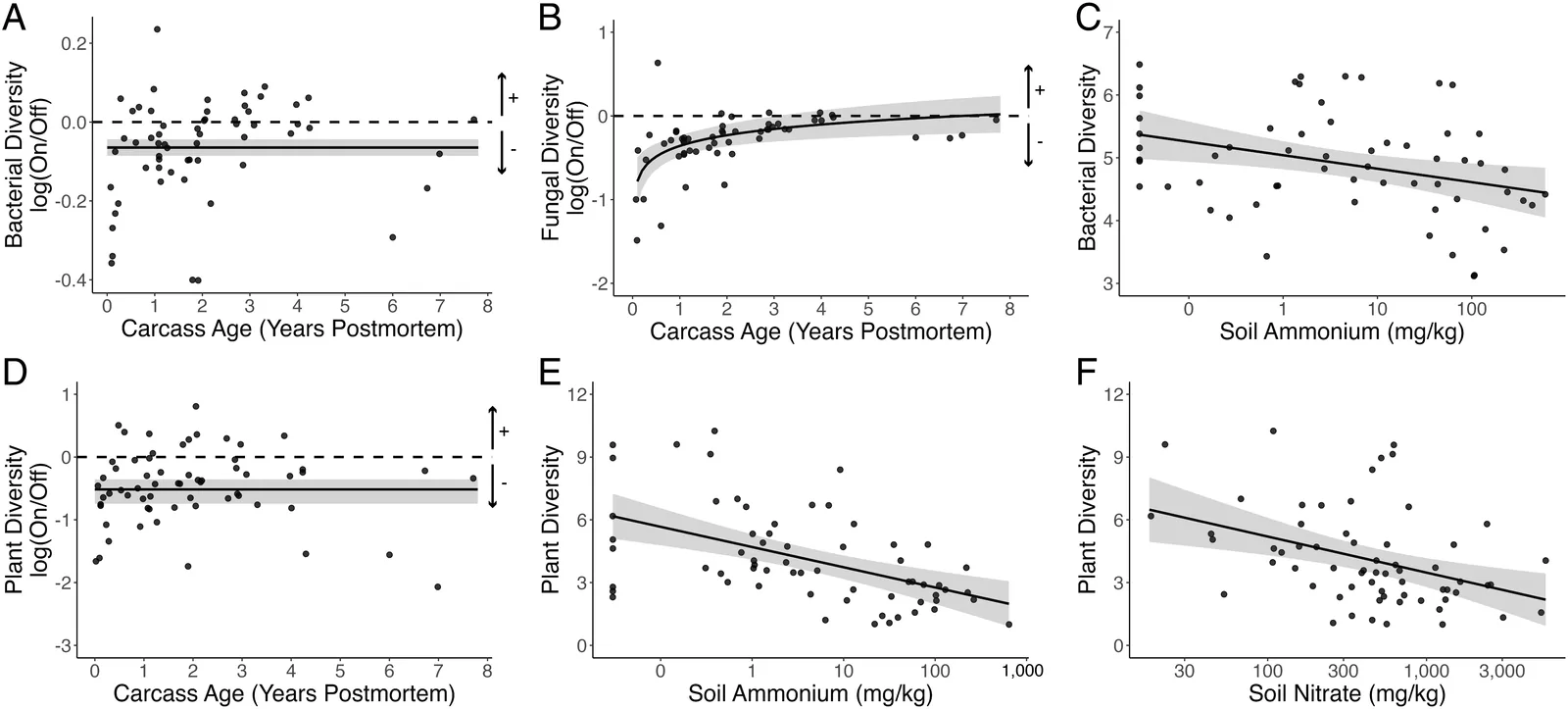
Impacts of elephant carcasses on below and aboveground biodiversity. (*A*) Bacterial and (*B*) fungal Shannon diversity were both reduced at elephant carcass sites. Fungal diversity recovered over time, while bacterial diversity did not. (*C*) Bacterial diversity was strongly negatively correlated with soil ammonium concentration. (*D*) Plant diversity was also strongly suppressed on carcass sites relative to off, a trend that persisted even at the oldest carcasses and was negatively correlated with (*E*) ammonium and (*F*) nitrate concentrations. See Fig. 1 for figure characteristics. For reference, an LRR of 1 means the value on the carcass is ~2.7× greater than off. Points in *C*, *E*, and *F* represent individual measurements and were taken only from on carcass sites. For bacterial and fungal samples, the on carcass measurements are samples taken at 0.5 m, while the on carcass value for plants is the mean of samples taken at 0.5 and 2.5 m. Lines show predictions calculated from the linear mixed effects models, and only significant relationships are shown on the plots as in Fig. 1.

Microbial diversity was significantly suppressed at carcass sites, likely due to high soil ammonium (*SI Appendix*, Table S2). Bacterial and fungal Shannon diversity were both significantly suppressed in carcass soils (lmm: *q* = 0.009 and 0.004; Fig. 2 *A* and *B*). Bacterial diversity remained suppressed and did not change with carcass age (*q* = 0.526), while fungal diversity recovered over time (*q* = 0.004). Neither bacterial nor fungal diversity showed differences with soil type (*q* = 0.670 for both), nor were there significant carcass age × soil type interactions (*q* = 0.117 and 0.670, respectively). Bacterial diversity was negatively correlated with soil ammonium concentration (q = 0.020; Fig. 2*C*), but fungal diversity was not (q = 0.649). Neither bacterial nor fungal diversity were correlated with soil nitrate (*q* = 0.527 and 0.517, respectively) or soil P (*q* = 0.276 and 0.527, respectively; *SI Appendix*, Table S3).

**Fig. 3.**
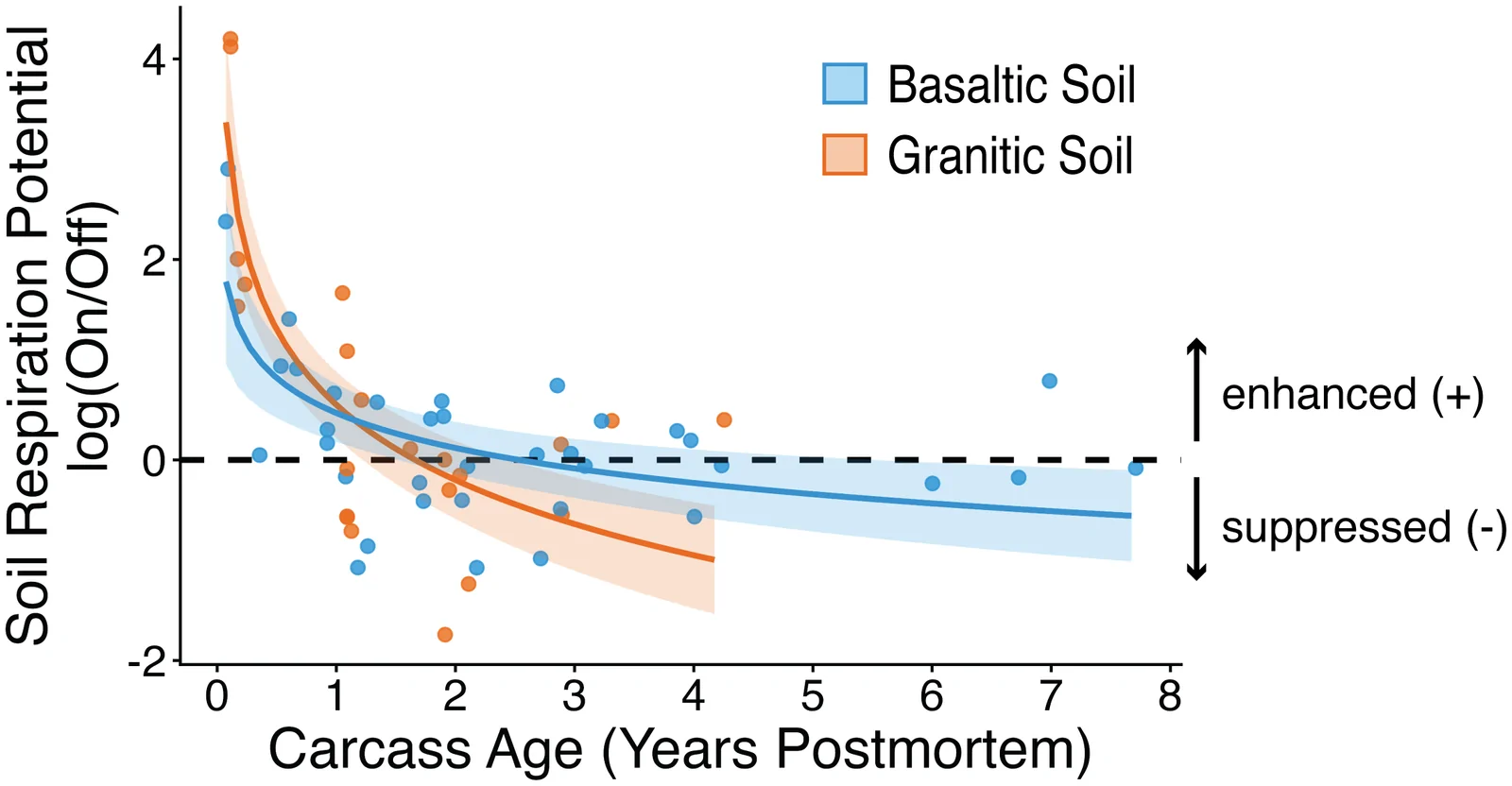
Impacts of elephant carcasses on soil respiration potential. Soil respiration was elevated on carcass sites relative to off carcass sites for about 1 y postmortem, decreasing more rapidly on granitic soils relative to basaltic soils. See Fig. 1 for figure characteristics. For reference, an LRR of 4 means the value on the carcass is ~55× greater than off, and LRR of 2 means the value on carcass is ~7× greater. Lines show predictions calculated from the linear mixed effects model, which included the log of carcass age, soil type, and a log of carcass age × soil type interaction as covariates. Thus, the different plots for granitic and basaltic soils represent the significant log of carcass age × soil type interaction.

Despite the decrease in microbial diversity, soil respiration potential was elevated on carcass sites (lmm: *q* = 0.006), with the effect decreasing over time such that it returned to off carcass levels between 1 to 2 y postmortem (*q* = 0.004; Fig. 3 and *SI Appendix*, Table S4). There was a significant carcass age × soil type interaction in which the effect of the carcass on soil respiration potential was stronger but also decreased more rapidly in granitic soils (*q* = 0.006). On both soil types, respiration potential initially increased but then appeared to be suppressed at older carcasses.

### Megacarcasses Suppressed Plant Community Diversity.

Elephant carcasses also strongly suppressed plant diversity relative to the surrounding savanna (*SI Appendix*, Table S2). Shannon diversity was significantly suppressed on carcass sites (*q* = 0.004) and remained so throughout the time series (*q* = 0.263; Fig. 2*D*). Plant diversity was negatively associated with both ammonium (*q* = 0.004; Fig. 2*E*) and nitrate (*q* = 0.004; Fig. 2*F*) in soils but was not significantly correlated with soil P-Bray 1 (*q* = 0.875).

### Megacarcasses Attracted Herbivores, Increased Herbivory, and Suppressed Plant Biomass.

**Fig. 4.**
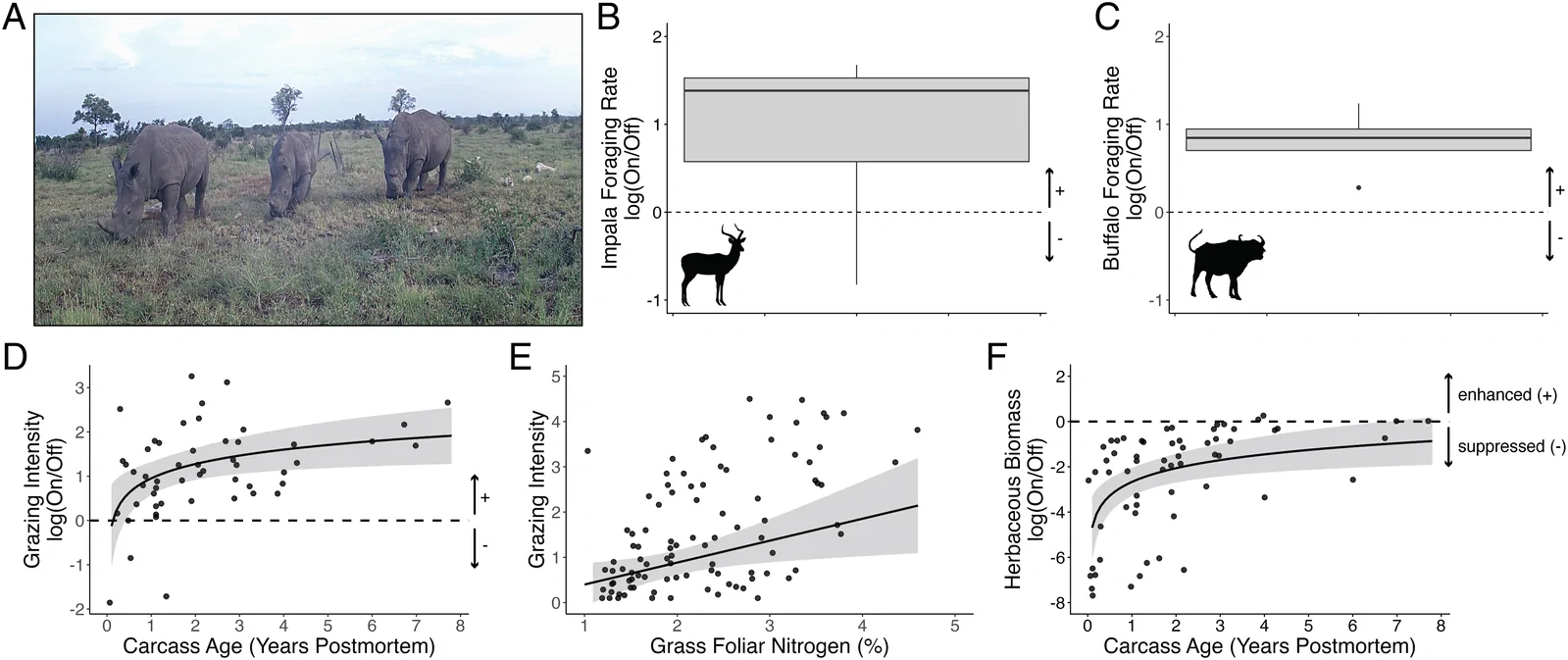
Impacts of elephant carcasses on savanna herbivore communities. (*A*) Mammalian herbivores, including white rhinoceros, were observed foraging on elephant carcass sites. The two most abundant herbivores, (*B*) impala and (*C*) buffalo, had significantly greater foraging rates on carcass sites relative to off. (*D*) Grazing intensity was elevated at carcass sites and increased significantly with carcass age. (*E*) Grazing intensity was also strongly positively correlated with the elevated foliar N on carcass sites. (*F*) Nutrient-driven increases in grazing contributed to reduced herbaceous plant biomass, which gradually recovered over the 7+ y period. On the boxplots (*B* and *C*), boxes show the interquartile range, the middle line indicates the median, whiskers extend up to 1.5× the interquartile range, and points are shown for outliers that fall >1.5× outside the interquartile range. For *D*–*F*, see Fig. 1 for figure characteristics. For reference, an LRR of 3 means the value on the carcass is ~20× greater than off.

Megacarcasses attracted a range of mammalian herbivores, such as elephant, impala (*Aepyceros melampus*), zebra (*Equus burchelli*), buffalo (*Syncerus caffer*), kudu (*Tragelaphus strepsiceros*), warthog (*Phacochoerus africanus*), and occasionally southern white rhinoceros (*Ceratotherium simum simum*) (Fig. 4*A*). The two most abundant herbivore species by far were impala (N = 22,827) and buffalo (N = 1,628), each of which had foraging rates ~3× greater on carcass sites relative to off carcass sites (*P* = 0.012 and 0.027, respectively; Fig. 4 *B* and *C*). These results were reinforced by results for grazing intensity, which was significantly elevated on carcass sites (*q* = 0.004), and the effect actually increased with carcass age (*q* = 0.013; Fig. 4*D* and *SI Appendix*, Table S5).

These increases in herbivore activity were likely driven by increased foliar nutrients in grasses at carcass sites. Foliar percent N and δ^15^N were both significantly elevated in grasses growing on carcass sites (*q* = 0.004 for both; *SI Appendix*, Fig. S3 and Table S6) and remained elevated regardless of carcass age (%N: *q* = 0.285; δ^15^N: *P* = 0.670). Foliar %N was ~1.5× greater on carcass sites compared to off. Foliar %N was also greater in grasses growing in basaltic soils compared to grasses in granitic soils (*q* = 0.013), but there was no significant carcass age × soil type interaction (*q* = 0.527).

Grazing intensity was positively correlated with foliar %N (*q* = 0.037; Fig. 4*E* and *SI Appendix*, Table S7), indicating preferential consumption by herbivores of the N-rich grasses. The impact of increased herbivory on carcass sites likely contributed to reduced plant biomass. Herbaceous plant biomass was strongly suppressed on carcass sites (*q* = 0.004) and slowly recovered over time but remained suppressed for most of the chronosequence (*q* = 0.004; Fig. 4*F* and *SI Appendix*, Table S2). Thus, elephant carcasses persisted as herbivory hotspots with low plant biomass for at least 7 y postmortality.

## Discussion

Terrestrial megafauna have shaped ecosystems for millions of years, influencing nutrient cycling, fire regimes, plant communities, and ecosystem transitions (9, 21–23). Yet, most research has focused on the effects of living animals, while the ecological legacies their carcasses create after death remain largely unexplored. Our results show that elephant carcasses generated persistent nutrient hotspots that suppressed the diversity of soil bacteria and fungi as well as the diversity of plants, creating coldspots of biodiversity on the landscape that lasted for years. The nutrient legacies of megacarcasses also created hotspots of ecosystem processes. Microbial respiration in the soil remained elevated for over a year, while increased herbivory from mammalian herbivores attracted to the N-rich grasses at carcass sites persisted for the duration of our time series. By linking nutrient inputs from megacarcasses to impacts on both community dynamics and ecosystem processes, our work revealed the persistent ecological legacies that terrestrial megafauna create after they die, a previously unappreciated dimension of how megafauna function as ecosystem engineers.

### Megacarcasses Suppressed Belowground Diversity While Increasing Rates of Microbial Processes.

Nutrient inputs from megacarcasses reduced soil microbial diversity (Fig. 2 *A* and *B*) while increasing soil microbial respiration (Fig. 3). Other studies similarly show that the carcasses of other terrestrial mammals (e.g., moose, elk) can decrease soil microbial richness (13) while increasing soil microbial biomass (5) and respiration (13). However, because of their massive size, elephant carcasses had longer-lived impacts on soil microbial communities than other mammals. Elephant carcasses resulted in increased soil respiration for ~1 to 2 y compared to <6 mo for elk and bison (13), and elephant carcasses suppressed microbial diversity for nearly twice as long as moose carcasses (5).

The suppression of soil bacterial diversity by elephant carcasses appeared to be due to the persistently high concentrations of ammonium in the soil (Fig. 2 *A* and *C*). The suppression of diversity by ammonium could be driven by several different mechanisms. First, the conversion of ammonium to nitrate increases soil acidity, which can contribute to losses in diversity (24, 25). Acidification due to nitrification could have influenced diversity at carcass sites, as soil pH appeared consistently lower at carcasses on basaltic soils and was lower at older carcasses on granitic soils after initially increasing at younger carcasses (*SI Appendix*, Fig. S2 and Table S1). Although nitrification can be suppressed at carcass sites due to soil hypoxia from the influx of fluids (14), this suppression does not appear to be occurring, for any length of time, at elephant carcass sites, given the extremely high soil nitrate concentrations that occur during the 1 to 2 y age range (Fig. 1 *E* and *F*). Second, high ammonium levels may create conditions that favor copiotrophic microbes over oligotrophic microbes, facilitating the dominance of ammonium-tolerant microbes (26, 27). In addition to high ammonium, carcasses could change other abiotic factors in the soil environment (e.g., hypoxia, increased temperature, substrate quality, compaction) that we did not measure but that can also affect microbial community composition (28, 29). Finally, reductions in soil microbial diversity may reflect feedback between belowground and aboveground processes, as reductions in plant biomass and diversity may result in the loss of plant-associated microbes in the soil as the plants change their investment in belowground partners (30).

The long-term elevation of soil P with no decline over time across both soil types is an important result in a system that is typically P-limited (31). One potential mechanism driving the persistence of P at carcass sites is that P is relatively immobile in soils, particularly in acidic soils, as decreased pH results in increased binding to minerals such as iron and aluminum, the availability of which increases at low pH, thereby decreasing availability to plants (14, 32). While soil pH did not differ significantly on vs. off carcass sites, it was relatively low in soils in the KNP, especially in granitic soils, where pH was consistently less than six (*SI Appendix*, Fig. S2). Thus, binding of carcass-derived P to soil minerals could cause P to persist longer at carcass sites, especially if plant biomass (and therefore uptake) is low for extended periods, as our data show. A second potential mechanism that creates persistent P hotspots is that elephant bones likely leach P as they weather, acting as a slow-release fertilizer at carcass sites. For example, fresh bones from wildebeest carcasses leach substantially greater N than P, but that ratio shifts to favor P in leachate as the bones age (33). The leaching of P from bones would require a persistent aggregation of bones, meaning that scavenging activity would likely determine the long-term trends in P enrichment, with higher scavenging rates potentially short-circuiting long-term P enrichment. For example, the nutrient impacts of wildebeest carcasses last longer when in scavenger-exclusion cages (34). Alternatively, visiting scavengers deposit waste inputs that could enhance P-enrichment, especially scavengers such as hyenas that consume bones and therefore have high concentrations of P in their dung (35). A more explicit examination of the impact of scavengers on megacarcass nutrient dynamics would be a fruitful area of future research.

The magnitude and duration of megacarcass nutrient legacies were modulated by soil type. Soil nitrate (Fig. 1*F*), phosphorus (Fig. 1*H*), pH (*SI Appendix*, Fig. S2), and respiration potential (Fig. 3) all showed a pattern in which the initial impact of the megacarcass was greater on granitic soils. This difference was likely due to differing physical and chemical properties of the two soil types. Basaltic soils are typically more nutrient-rich than granitic soils (36, 37), with total C concentrations 2 to 4× and total N concentrations 2 to 6× higher than in granitic soils (38). Thus, the higher baseline nutrient concentrations in basaltic soils may make the initial impact of the carcass less stark than in granitic soils. However, basaltic soils are also clay-rich, providing a larger, charged surface area that makes adsorption more efficient and leaching less likely than in granitic soils (37, 39). Basaltic soils also have a higher proportion of micropores, slowing down nutrient turnover and increasing water retention relative to the sandier granitic soils. Combined, these factors likely contributed to more persistent legacies on basaltic soils from the nutrient pulses that megacarcasses create (38, 39).

### Megacarcasses Suppressed Plant Diversity and Biomass While Facilitating Herbivory.

Elephant carcasses suppressed plant diversity for at least 7 y postmortem, with little sign of recovery over that period (Fig. 2*D*). The duration of this impact is substantial in comparison with carcasses of smaller species, which exhibited either no change in plant diversity [wildebeest (34)], increases in exotic species richness but not overall diversity [kangaroo (17)], or brief (~1 y) suppression of plant diversity followed by quick recovery [bison, cattle, deer (16)].

Multiple mechanisms were likely driving the effects of elephant carcasses on plant diversity, with the relative importance of each changing with carcass age. Plant diversity was negatively correlated with soil concentrations of ammonium (Fig. 2*E*) and nitrate (Fig. 2*F*), both of which were highly elevated at carcass sites (Fig. 1 *C*–*F*). Soil ammonium concentrations were especially extreme at the freshest carcass sites, reaching concentrations almost 3,000× those found off carcass sites (Fig. 1 *C* and *D*; log response ratio of eight indicates ~3,000× baseline). These high levels of ammonium can be toxic for plants (40), which is likely one reason why plant diversity and biomass were suppressed at fresh carcasses (Figs. 2*D* and 4*F*). During these early stages, the overwhelming effects of high nutrient concentrations on plant physiology likely played a strong role in suppressing diversity, with competition for resources relatively unimportant.

As carcasses age, competition among plants could become more important as high soil nutrients facilitate competitively dominant species, especially species with high ammonium tolerance. The conversion of ammonium to nitrate by soil microbes could facilitate this movement toward competition, as the presence of nitrate alleviates ammonium toxicity for some plant species (41, 42). At older carcass sites, plant biomass had recovered significantly (Fig. 4*F*) but nutrients were still high (Fig. 1 *C*–*H*) and diversity was still low (Fig. 2*D*), suggesting that plants that could take advantage of these nutrient rich soils and tolerate elevated grazing levels (Fig. 4 *D* and *E*) may have been outcompeting other species. Further research on older carcasses would be beneficial for elucidating these patterns of plant diversity in the long-term, as we expect that continued herbivory and decreased soil ammonium levels would continue to influence plant diversity past the 7.7 y measured in this study.

Feedback between plant and herbivore communities may be driving patterns of plant biodiversity, plant biomass, and herbivore foraging activity. Mammalian herbivores are attracted to high-nutrient foliage and often seek out nutrient hotspots (43), such as grazing lawns (44) and termite mounds (45). Grazing surveys at 36 carcasses and more limited camera trapping at eight carcasses both showed elevated grazing on N-rich grasses at carcass sites (Fig. 4 *A*–*E*). This more intense herbivory likely contributed to the strong reductions in herbaceous plant biomass at carcass sites (Fig. 4*F*), which persisted for at least the first 5 y postmortem. Moreover, the prolonged elevation of foliar N levels across the time series may represent a positive feedback started by elephant carcasses and maintained by herbivores. Given that herbivores were attracted to carcass sites and that grazing remains elevated even at the oldest carcasses, their increased visitation likely increased nutrient deposition from dung and urine (46, 47) that further enriched the site and attracted more herbivores. Thus, elephant carcasses potentially initiate a positive feedback cycle, similar to grazing lawns (48, 49), where megacarcass sites become long-term hotspots of nutrient cycling and ecological activity. Feedback from herbivore visitation could play an important role in extending the duration of megacarcass impacts on savanna ecosystems, and experimental manipulations and/or long-term monitoring would be key for testing these positive feedback.

Although we did show strong and persistent effects of elephant carcasses on nutrients, diversity, and ecosystem processes, there was significant variance around many of the effects. For example, there was significant variability in the effect of carcasses on plant and bacterial Shannon diversity and soil ammonium, with carcass age and soil type explaining only some of the variance (e.g., R^2^ = 0.05 for plant Shannon diversity, 0.18 for bacterial Shannon diversity, and 0.15 for soil nitrate). A number of unmeasured factors such as elephant size and sex, rainfall differences, and scavenger density likely contributed to variation among sites. Some of the variability is likely also due to the natural heterogeneity of savanna ecosystems. However, we tried to minimize this variability as much as possible by collecting data from carcass sites only in open savanna and woodland habitats, the most common habitats in the KNP, and avoiding carcasses near riparian areas, in sodic sites, or on hillslopes. We also sampled such that carcass ages were spread across the landscape, with both fresh and old carcasses taken across the latitudinal gradient and on both soil types. Yet, background variation in soil nutrients, water drainage, plant communities, scavenger abundance, herbivore abundance, etc. likely contributed to variation in the impacts of elephant carcasses.

Several of these unmeasured sources of variation in the impact of elephant carcasses provide important avenues for future research. For example, the season in which an elephant dies could shape its ecological legacy. Nutrient inputs into dry soils are less mobile than those in wet soils (50), suggesting that the nutrient legacies from carcasses created in the dry season may persist for longer. Further, an elephant carcass deposited in the dry season may minimally impact the plant community, but a carcass deposited in the wet season, plus the scavenging activity at the carcass, could create a larger impact on plant diversity. Thus, the season of death is potentially an important contributor of variation in carcass site dynamics and would be an important focus for future studies.

### Megacarcasses Created Unique, Integrated, and Persistent Ecological Legacies.

The size of elephant carcasses makes them functionally distinct from smaller carrion. The impact zone of an elephant carcass has a radius of ~5 m with an ~80 m^2^ footprint (6). In contrast, even relatively large mammals such as bison (16) and muskoxen (*Ovibos moschatus*) (51) generate smaller, localized zones of disturbance with radii of 0.5 and 2 m that correspond to footprints of <1 m^2^ and ~12 m^2^, respectively. Thus, the spatial influence of elephant carcasses can be well over an order of magnitude larger than that of other (even relatively large) mammals. Elephant carcasses are also unique in that they take much longer to decay than smaller carrion; in the absence of scavengers, soft tissues may take up to 2 y to decompose, with bones remaining for decades (52). Conversely, smaller mammals such as rabbits take only about 20 d to reach skeletonization (12). Moreover, smaller-bodied animals experience greater relative mass loss due to scavengers (14, 53), meaning that the size of megacarcasses makes it more likely that substantial amounts of nutrients percolate into the soil layer in a localized area. Yet, despite the enormous size of elephants, their carcasses rarely have time to decompose in KNP due to high scavenger densities [e.g., lions, *Panthera leo*; vultures, *Gyps africanus*; hyenas, *Crocuta crocuta* (54, 55, 56)], which quickly consume meat and disperse bones. The robust scavenging guild in KNP means that we are likely significantly underestimating the strength and duration of the ecological legacies of megacarcasses in ecosystems that have lower scavenger densities. Thus, further research into the impacts of scavenging on carcass nutrient dispersal is needed to understand the full impacts of megacarcasses on savanna ecosystem processes (57–59).

The legacy effects of megacarcasses in savanna ecosystems are extreme in both magnitude and duration relative to smaller carrion. Moose carcasses yielded mean soil inorganic N (ammonium + nitrate) concentrations of ~300 mg/kg (max ~450 mg/kg) at their peak (5), while kangaroos resulted in maximum soil ammonium concentrations of ~80 mg/kg and nitrate concentrations of ~225 mg/kg [~305 mg/kg inorganic N (12)]. In contrast, we measured maximum soil ammonium concentrations at elephant carcasses of 640 mg/kg and nitrate concentrations of 5,622 mg/kg, for a maximum total inorganic N value of 6,262 mg/kg (~14× moose and 20× kangaroos). Maximum plant-available P concentrations in elephant carcass soils were ~6× greater than those found in kangaroo carcass soils [936 vs. 150 mg/kg (12)]. Moreover, inorganic N concentrations in soils below moose carcasses converged with control soils by 28 mo postmortem, and P converged by 40 mo (5). In South African savannas, blue wildebeest carcass sites showed elevated soil nutrients for only 6 to 18 mo, while plant biomass and diversity recovered after just 1 y (60). In contrast, we show that soil ammonium, nitrate, and plant-available P were all still elevated at elephant carcass sites 7+ y postmortem, while plant biomass and diversity were suppressed for 4+ y and 7+ y, respectively, emphasizing the unique time scale of the legacies of megacarcasses.

The nutrient legacies of megacarcasses appear to last longer in plants than in soils; while soil N declined over time foliar N remained elevated across the time series. Studies of the largest carrion that are not considered megafauna (e.g., bison, elk, muskoxen, moose) showed similar patterns with elevated foliar N concentrations at carcass sites lasting up to 2 to 5 y (5, 13, 51). Yet, at elephant carcasses, foliar N had not begun to diminish even after 7+ y (*SI Appendix*, Fig. S3). This persistently elevated foliar N (and δ^15^N) may be indicative of a positive feedback cycle in which the nutrient pulse from carcasses attracts herbivores. Increased foraging and defoliation, in conjunction with continued inputs of nutrients via urine and dung, may thus contribute to the persistence of the nutrient hotspot (61).

### Conclusion.

Much attention has been given to the implications of megaherbivore population declines due to their impacts while alive (19, 62). Here, we demonstrate that their ecosystem engineering effects continue years after death, as their megacarcasses create integrated ecological legacies that affect nutrient cycling, biodiversity, and ecosystem processes. These impacts are intense and long-lasting, making them fundamentally distinct from smaller carrion. Moreover, the impacts of terrestrial megacarcasses, which suppress diversity across three different domains of life, are fundamentally opposite to the biodiversity-increasing impacts of whale falls (7, 8), demonstrating that terrestrial megacarcasses are unique even compared to their marine counterparts. Given that KNP has a population of ~30,000 elephants with about a 2% mortality rate (63), roughly 600 elephants die in KNP every year. Our data show that the ecological legacies that these megacarcasses create on belowground and aboveground processes last at least 7 y and likely much longer, given that we saw no diminution of the legacies at many of the older carcass sites. Thus, at any one point in time there could be ~4,000 elephant carcasses in KNP that are influencing the ecology of this ecosystem with varying intensities and durations of their legacies.

Our data come from one of the few remaining refuges for megaherbivores on Earth. Yet, prior to the Pleistocene extinctions, megafauna such as elephants, rhinoceros, and their relatives were globally abundant in ecosystems ranging from the tropics to the poles (64). Thus, megacarcasses and their ecological legacies would once have been key elements to the ecology of many of Earth’s ecosystems. The near planet-wide absence of terrestrial megafauna now likely represents a near functional extinction of the ecological processes that are driven by megacarcasses. We are only beginning to understand what losing large species has meant for the transport and fate of nutrients across ecosystems (21, 62, 65) with little known about how megacarcasses might affect those dynamics. The unique ecological role of megacarcasses is now found in only a limited number of Earth’s ecosystems, where it is at risk due to large-scale megafaunal declines and extirpations. Given the magnitude, duration, and ecosystem-spanning integration of these impacts, the loss of megacarcasses could represent a fundamental shift in the structure and function of ecosystems across the globe.

## Materials and Methods

### Study System.

We performed this research in Kruger National Park (KNP), South Africa (24.996° S, 31.592° E). The two dominant soil types in KNP are granitic soils (Inceptisols) and basaltic soils (Vertisols) (66). Basaltic soils are clay-rich, comprised of ~37% clay and 32% sand, where the granitic soils contain 5% clay and 85% sand (39). Thus, granitic soils tend to leach water more quickly and therefore have lower concentrations of water-soluble nutrients compared to basaltic soils (39). The landscape of KNP is a mix of savanna grasslands and broadleaf woodlands, with an overstorey dominated by trees from the genera *Combretum* and *Dichrostachys* and trees formerly known as acacias (e.g., knobthorn, *Senegalia nigrescens*) in the southern part of the park and an overstorey dominated by mopane (*Colophospermum mopane*) in the north. The park hosts a large suite of African savanna herbivores such as zebra, buffalo, blue wildebeest, white rhinoceros, and impala (54). Importantly, KNP has ~30,000 African savanna elephants, with a mortality rate of ~2% [~600 elephant carcasses created per year; (63)].

The exact georeferenced locations of elephant carcasses were determined via a database of carcass locations maintained by KNP as well as via consultation with park management. Elephant mortality was from a variety of causes including old age, illness, injury, and territorial disputes. Managers typically locate the carcasses through ground patrols, aerial surveys, and by tracking aggregations of scavengers such as vultures, hyenas, and lions. Thus, there may be some detection bias toward regions that are relatively more open and closer to roads. We identified 40 megacarcass sites that were divided between granitic (N = 13) and basaltic (N = 27) soils (Fig. 1*A*). To help ensure carcasses were comparable, we focused on carcasses that were in open woodland and savanna habitat with relatively flat aspects. Further, we avoided sampling carcasses that were located in riparian areas, sodic sites, or on hillslopes. Eight of the 40 sites had been initially sampled during the 2023 wet season and published in previous work (6), although the previously published data are not included in the analyses here. The rest of the carcass sites were newly identified. In 2024, we sampled 27 sites (N = 12 on granitic and 15 on basaltic soils). In 2025, we performed repeat sampling on 24 of those sites and added 13 new sites (N = 64 carcasses sampled across both years, with 40 unique sites; *SI Appendix*, Table S8). In both years, sampling was performed toward the end of the wet season (late February to early April). Because some fresh sites had minimal plant material, the sample sizes for plant-focused analyses vary and are included in each of the relevant supplemental tables. All megacarcasses were from adult elephants and had a range of time since mortality of 8 to 2,814 d (0 to 7.7 y) at the time they were sampled (*SI Appendix*, Fig. S4). The center of each megacarcass site was determined to be the center of the impact zone from the carcass, which was typically characterized by an area of bare soil that contained bones, bone fragments, and/or gut contents (Fig. 1*B*).

### Effects of Megacarcasses on Soil and Foliar Nutrients.

To test the impacts of elephant carcasses on soil and foliar nutrient concentrations, we collected soil and grass leaf samples from the 40 elephant carcass sites. We placed a short metal post at the center of each carcass site and ran one 15 m transect in each of the four cardinal directions radiating from the center. Prior research indicated that the effects of elephant carcasses on soil and foliar nutrients do not persist farther than ~5 to 10 m from the center of the carcass site (6). Thus, we collected samples from 0.5 m and 2.5 m away from the center of the carcass to represent the effect of the carcass (e.g., “on carcass” samples) and collected samples from 15 m away from the center to represent baseline conditions (e.g., “off carcass” samples). At the three points along each transect (0.5, 2.5, and 15 m), we used an auger to collect soil samples to a depth of 10 cm (11, 18) then sieved samples through a 5 mm metal sieve that we cleaned in between samples with 70% ethanol. At the same three distances on each transect, we collected green leaves from one grass species (preferentially *Urochloa trichopus* or *Megathrysus maximus* if *U. trichopus* was not abundant at a site). We pooled and homogenized samples from each transect to obtain one composite soil and one composite leaf sample per distance per carcass site (e.g., one soil and one grass sample at 2.5 m distance). We stored the soil samples in plastic bags at room temperature prior to analysis. We dried the leaf samples in a drying oven at 65 °C for 48 h and stored the dry plant material in paper bags prior to analysis. Concentrations of soil ammonium and nitrate were determined using a 1:2 soil:water extraction method, soil plant-available P was quantified using a P-Bray 1 extraction, and soil and foliar δ^15^N were measured using stable isotope analysis (*SI Appendix, Text S1*).

### Effects of Megacarcasses on Soil Microbial Diversity and Respiration.

We assessed soil bacterial and fungal diversity via 16S rRNA and ITS gene amplicon sequencing, respectively (*SI Appendix, Text S2*). We quantified soil respiration within 24 h of sampling using a soil incubation method (*SI Appendix, Text S3*).

### Effects of Megacarcasses on Plant Diversity and Biomass.

We characterized plant community composition at each of the elephant carcass sites. For all four transects at each site, we placed a 1 × 1 m quadrat on the right side of the transect tape centered at 0.5, 2.5, and 15 m. We identified every plant within each quadrat to species level (67, 68), visually estimated its percent aerial cover within the quadrat to the nearest 1%, and categorized each species as grass, forb, or woody (69). To quantify herbaceous plant biomass, we took vegetation height measurements at 1 m intervals along each transect using a disc pasture meter (DPM). When bones or small shrubs prevented the disc from settling on the vegetation, we performed the measurement on the left side of the transect. Occasionally it was necessary to move slightly to the left or right of the transect to obtain an accurate measurement, always moving laterally to maintain the correct distance from the carcass center. We converted DPM measurements to plant biomass using a KNP-specific calibration for DPM heights (70).

### Effects of Megacarcasses on Herbivore Abundance and Herbivory.

We tested the impacts of elephant carcasses on the abundance and foraging behavior of mammalian herbivores using grazing surveys and camera traps. To quantify herbivore grazing, we surveyed the grazing intensity at 1 m intervals along each transect. We selected the grass tuft closest to the transect tape and gave it a grazing intensity score ranging from 0 (completely intact) to 5 (completely grazed to the soil surface) (45). To quantify the abundance of herbivores and their foraging behavior at carcass sites, we established camera traps at eight paired carcass (camera aimed at the center of carcass site) and background (~20 m North of each carcass center) sites at varying intervals between August 2023 and March 2025 (*SI Appendix*, Table S9 and *Text S4*). Within a site, both carcass and background cameras ran concurrently. The duration of camera trap footage varied among sites (range = 39 to 224 d, mean = 129 d) due to frequent destruction of camera traps by elephants and hyenas. To determine how elephant carcasses affected mammalian herbivory, we quantified the species identity and number of individuals recorded on the camera trap footage for the eight paired carcass and background camera traps (*SI Appendix*, Table S9; N = 1,031 total days of footage). We also recorded whether any individuals in a given image were foraging.

### Statistical Analyses.

All statistical analyses were run in R version 4.3.1 (71).

To test the impacts of elephant carcasses on soil and plant nutrients and soil pH, we first calculated the mean nutrient concentrations for each soil and foliar nutrient at the 0.5 and 2.5 m distances for each site, treating the mean of these two distances as the on carcass sample at that site. Samples from the 15 m distance represented background off carcass concentrations for each site. We calculated the log response ratio (LRR) for on carcass vs. off carcass values (LRR = ln(on carcass/off carcass) for each site. Then for each metric (i.e., soil ammonium, soil nitrate, soil P-Bray 1, soil pH, foliar %N, and soil and foliar δ^15^N), we ran separate linear mixed effects models using the lme4 package (72) with LRR as the response variable and the log of carcass age, soil type, and the log of carcass age × soil type interaction as fixed effects. The carcass site was included as a random effect to account for the fact that we sampled some carcasses in both 2024 and 2025.

To determine how elephant carcasses affected soil microbial diversity, we calculated bacterial (16S) and fungal (ITS) Shannon diversity in phyloseq (73) at the 0.5 m (on carcass) and 15 m (off carcass) distances. DNA was not extracted from the soil samples from 2.5 m so we did not pool the 0.5 m and 2.5 m distances as for other analyses. Thus, soil microbial samples likely represent more extreme conditions than the measurements for soil and foliar nutrients and plant diversity. We ran the same models as above separately for bacterial and fungal diversity, with LRR as the response variable and the log of carcass age, soil type, and the log of carcass age × soil type interaction as fixed effects. The carcass site was included as a random effect. Next, we tested for correlations between soil nutrient concentrations and microbial Shannon diversity. Because soil nutrient values clustered at very low values off carcass sites relative to the wide range of values on carcass sites, we filtered the data to just on carcass samples to focus the model on the diversity vs. nutrient relationships. We then ran separate linear models correlating bacterial and fungal Shannon diversity with each soil nutrient (i.e., ammonium, nitrate, plant-available P) with carcass site as a random effect.

To determine the impacts of elephant carcasses on plant communities, we analyzed plant species diversity and herbaceous plant biomass. We filtered the data to include only grasses and forbs to focus on the herbaceous plant community. We calculated the mean species percent cover values of each species at 0.5 and 2.5 m to obtain the on carcass values and used the 15 m cover values as off carcass values. We also calculated the mean across the four transects to obtain one on carcass and one off carcass value per carcass site. We calculated plant Shannon diversity using the HillR package (74) then calculated the LRR for on vs. off carcass and ran a linear model as described above. Next, we tested for correlations between soil nutrient concentrations and plant Shannon diversity by filtering the data to just on carcass samples. We then ran separate linear models correlating Shannon diversity with each soil nutrient (i.e., ammonium, nitrate, plant-available P). Because DPM measurements were taken at 1 m intervals along each transect, we measured herbaceous plant biomass by calculating the mean DPM measurements from 0 to 2 m distances on every transect to represent the on carcass value and the mean from 13 to 15 m distances to represent the off carcass control. We calculated the LRR for each carcass and used a linear model, as described above.

Finally, we tested the impacts of elephant carcasses and their associated nutrient inputs on herbivory. We filtered the grazing intensity dataset to include only palatable grasses, which we defined as any grass species that had been grazed at least once in the dataset. We then calculated the mean of all the grazing scores at the 0 to 2 m distances on every transect to represent the on carcass value and calculated the mean scores from 13 to 15 m distances to represent the off carcass value. We calculated the mean across the four transects to get one on carcass and one off carcass grazing intensity score per carcass site. We calculated the LRR for on vs. off carcass for grazing intensity and used a linear mixed effects model to test the effects of the carcass as described above. We also ran a linear model correlating foliar N with grazing intensity, with carcass site as a random effect.

We then examined how elephant carcasses affected intensity of herbivory by using camera trap footage. We excluded elephants from these analyses because they visit carcasses for a variety of reasons related to social behavior rather than foraging (75), though we note that social visitation could still contribute to the nutrient feedback cycle via dung and urine deposition. To determine whether herbivore foraging was elevated at carcass sites, we filtered the dataset to buffalo (N = 1,628 individuals) and impala (N = 22,827 individuals), which together represented 91.6% of mammalian herbivore observations. For each of these two species, we calculated a foraging rate for each camera by dividing the number of individuals observed foraging by the number of days each camera was set. We calculated the LRR for on vs. off carcass sites and performed a *t* test to determine whether the LRR differed significantly from zero.

To account for the potential for false positives due to multiple hypothesis testing, we performed a global Benjamini-Hochberg correction (76) to the *P*-values for each parameter across all of our models. Throughout the results, we reported the more conservative Benjamini-Hochberg corrected *P*-values (*q-*values) rather than raw *P*-values, and decisions about which parameters to visualize in figures were also based on corrected *P*-values (*q*-values).

## Supplementary Material

Appendix 01 (PDF)

## Data Availability

All data and code needed to replicate our analyses and main figures are available at https://doi.org/10.5061/dryad.q2bvq841g (77). Carcass site metadata are included in the *SI Appendix*.
