# Supplementary material for: Nutrient legacies from elephant megacarcasses create coldspots of biodiversity and hotspots of ecosystem processes
Source: Proc Natl Acad Sci U S A. 2026 Oct 12;123(43):e2605332123. doi: 10.1073/pnas.2605332123 (PMC13715796; doi:10.1073/pnas.2605332123)
Supplement: Supplementary file 1 [file pnas.2605332123.sapp.pdf]

SUPPLEMENTAL MATERIALS

Text S1. Soil and foliar nutrient laboratory analysis methods

To determine concentrations of key soil nutrients, we sent 250 g of each soil sample to the Eco-Analytica laboratory at the North-West University in Potchefstroom, South Africa. Concentrations of soil ammonium $[NH_4]^+$ and nitrate $[NO_3]^-$ were determined using a 1:2 soil:water extraction method [1]. Samples were air dried and sieved through <2 mm mesh prior to chemical analysis and then extracted volume-on-volume 100 mg soil to 200 mL of deionized water. To determine soil ammonium concentrations, the solution was analyzed colorimetrically using a Systea EasyChem200 analyzer. To determine soil nitrate concentrations, ion chromatography was used (Metrohm 930 Compact Flex System). Ion concentrations were given as mg/L with a detection limit of 0.01 mg/L, which were converted to mg/kg by multiplying by 2 based on the 1:2 soil:water extraction ratio. To quantify plant-available P in soil, P was extracted from 4 g of soil using 30 mL of acid fluoride solution (P-Bray 1) [2]. Soil plant-available P concentration was analyzed colorimetrically using an EasyChem200 analyzer and expressed as mg/kg with a detection limit of 0.05 mg/kg.

To determine whether soil N was influenced by nutrients from the elephant megacarcass, we used stable isotope analysis, as higher values of $\delta^{15}N$ are typically indicative of animal-derived nitrogen [3]. $\delta^{15}N$ was measured from 10 g of each soil sample using a Vario Isotope Select Elemental Analyzer and an Isoprime Precisions isotope ratio mass spectrometer (IRMS) at the BIOGRIP laboratory within the Central Analytical Facility at Stellenbosch University. Samples were oven-dried at 60°C for 48 hours, milled to a fine powder using a Retsch MM400 mill (Germany), and then weighed prior to combustion at 950°C. Gasses were then reduced to undiluted N_2 in a reduction column held at 600°C. Along with the samples, a high organic carbon (HOC) standard (0.52 ± 0.02 %N) and two international reference standards (USGS40 and USGS41) were run for calibration. The N elemental content was

expressed relative to atmospheric N as N_2 $\delta^{15}N$ AIR (‰). Using the HOC standard, a quantification limit of 1 nA and a precision of ± 0.11‰ were determined.

To quantify the impact of elephant carcasses on foliar N concentrations, we used stable isotope analysis using IRMS as described above. Leaf %N and $\delta^{15}N$ were quantified using a sorghum flour standard (1.45 ± 0.25 %N) from Elemental Microanalysis Ltd (UK) and the same two international reference standards (USGS40 and USGS41) for calibration. The quantification limits for %N and $\delta^{15}N$ were 1.3% and 1 nA, respectively. Precision was 0.02% for %N and ± 0.08‰ for $\delta^{15}N$.

Text S2. Soil fungal and bacterial sequencing methods

We assessed soil bacterial and fungal diversity via 16S rRNA and ITS gene amplicon sequencing, respectively. From each composite soil sample, we passed 5 g of soil through a 1.5 mm mesh sieve. We then extracted DNA from 0.25 g soil using Qiagen DNeasy Power Soil Pro kits. Extracted DNA was frozen at -20°C until transport to Marquette University. Aliquots of each sample were then sent to Argonne National Labs for amplicon sequencing. We sequenced the prokaryotic V4 region of the 16S rRNA gene 515F - 806R and the ITS1f-ITS2 fungal regions using a 12-base barcode on the forward primer as outlined by the Earth Microbiome Project Protocol. The PCR mixture contained 9.5 µl of PCR water, 12.5 µl of Quantabio's Accustart II PCR ToughMix (2× concentration, 1× final), 1 µl of Golay barcode-tagged forward primer (5 µM concentration, 200 pM final), 1 µl reverse primer, (5 µM concentration, 200 pM final), and 1 µl template DNA. Thermocycler conditions were set to 94°C for 3 min, followed by 35 cycles at 94°C for 45 s, 50°C for 60 s, and 72°C for 90s, ending with a final extension of 10 min at 72°C. Samples were pooled into a single tube, quantified, and diluted to 2 nM. We then added 6.75 pM of 10% PhiX. Amplicons were sequenced on a 151-bp by 12-bp by 151-bp Illumina MiSeq run, and samples from both 2024 and 2025 were sequenced during the same sequencing run.

Raw sequence reads were demultiplexed using idemp [4]. Paired-end sequence reads were then processed in R [5] using the DADA2 pipeline [6]. Briefly, sequences were filtered and trimmed (truncated to minimum 145 bp with maximum error rate of 2 for forward and reverse reads), an amplicon sequence variant (ASV) table was constructed, and chimeras were removed. Taxonomy was assigned using the Silva database [7] for bacteria and the UNITE database [8] for fungi. Bacterial samples ranged from 1,489 to 201,197 reads per sample, while fungal samples ranged from 29 to 16,814 reads per sample (Figure S5). To account for this uneven read count, we rarefied each dataset to a sampling depth of 2,003 for 16S samples and 1,129 for ITS samples in an attempt to keep as many samples as possible, while still maintaining sufficient reads per sample. One bacterial sample and 5 fungal samples were

removed from the dataset during rarefaction due to insufficient sampling depth. We performed a sensitivity analysis to determine whether our results were impacted by rarefaction depth and determined that read coverage did not significantly affect our results (Text S5; Table S10-11).

Text S3. Soil microbial respiration methods

To quantify soil respiration potential, we used a soil incubation method [9,10]. We placed 5 g (± 0.2 g) of each soil sample into a 100 mL clear glass bottle, which was then sealed and flushed with CO_2-free air. We then incubated the bottles for one hour at 25°C and measured CO_2 concentrations using an LI-850 CO_2/H_20 infrared gas analyzer. Following incubation, we quantified sample dry weight by placing samples in a drying oven at 60°C until stable mass was achieved (24-48 hours). We then used the dry weights and the Ideal Gas Law to convert respiration measurements to CO_2 μg per hour per g of dry soil.

Text S4. Camera trap methods for quantifying herbivory

We tested the impacts of elephant carcasses on the abundance and foraging behavior of mammalian herbivores using camera traps. We established camera traps at eight paired carcass (camera aimed at center of carcass site) and background (~20 m North of each carcass center and facing away from the carcass) sites. The camera traps were a mix of CAMPARKCAM 36MP 2K Motion Activated Trail cameras (Model TC11) and Bushnell 16MP Trophy Cam HD Essential E3 trail cameras (2016 model). They recorded one image and one 5-second video each time motion was detected, with a 30-second lag between images. Camera traps were set up at varying intervals between August 2023 - March 2025, ranging from 39-224 days per site (Table S9). Within a site, both carcass and background cameras ran concurrently.

To determine how elephant carcasses affected mammalian herbivory, we quantified the species identity and number of individuals recorded on the camera trap footage for the eight paired carcass and background camera traps (Table S9; N = 1,031 total days of footage). We counted each set of animals within a given species recorded on the camera trap together as one “visit” and quantified the abundance of individuals for that visit. If less than one hour passed between images with the same species present, we considered it the same visit. If more than one hour passed between images, we counted the next set of individuals as a new visit following previous protocol standards [11,12]. We also recorded whether any individuals in a given image were foraging.

Text S5. Sensitivity analysis for 16S and ITS sequence rarefaction depth.

To test whether rarefaction depth impacted the results of our Shannon diversity models, we performed a sensitivity analysis. All of the following steps were performed separately for 16S and ITS sequencing data. First, we generated a suite of datasets in phyloseq [13] in which we started with the full sample set (N = 120 samples for 16S and 115 samples for ITS data) and dropped one sample at a time in order of increasing sequence depth, re-rarefying each time, until we reached a sample size of 60 samples (maximum rarefaction depth = 10,266 for 16S and 5,593 for ITS). We chose 60 samples because after calculating LRR, that would yield thirty observations for the model, which is the minimum number needed for a model with two parameters (carcass age, soil type) and their interaction. On each of those datasets, we ran linear mixed effects model with LRR of Shannon diversity as the response variable and the log of carcass age, soil type, and the log of carcass age × soil type interaction as fixed effects and carcass site as a random effect using lme4 [14]. Finally, we compared the coefficients and *p*-values for each model and parameter to determine whether rarefaction depth affected model outputs and interpretation of results.

Model results for bacterial and fungal Shannon diversity were not sensitive to rarefaction depth (Tables S10-11). For bacterial diversity models (Table S10), the intercept was consistently strongly significant ($p \leq 0.004$ for all models), and the soil type parameter was never significant. Log of carcass age began appearing significant at a rarefaction depth of 6,392, and log of carcass age × soil type interaction was occasionally significant, but the *p*-values were marginal, and few would have remained significant after the FDR correction. For fungal diversity (Table S11), intercept and log of carcass age were consistently strongly significant, while soil type and the log of carcass age × soil type interaction were never significant. Thus, for both bacterial and fungal Shannon diversity, the sensitivity analysis yielded results consistent with those reported in the main manuscript, demonstrating that rarefaction depth did not impact our results or interpretation.

References

1. C. Sonneveld, J. van den Ende, Soil analysis by means of a 1:2 volume extract. *Plant Soil* 35, 505-516 (1971).
2. FAO. “Standard Operating Procedure for Soil Available Phosphorus - Bray I and Bray II Method.” [Protocol] Available from https://openknowledge.fao.org/server/api/core/bitstreams/1e4693b1-9ee2-4e29-91d9-e2f310a8c384/content. (2021)
3. D. M. Post, Using stable isotopes to estimate trophic position: models, methods, and assumptions. *Ecol.* 83, 703-718 (2002).
4. Y. Wu. “Idemp: barcode demultiplex for Illumina I1, R1, R2 fastq.gz files.” [Software]. Available from https://github.com/yhwu/idemp. (2025)
5. R Core Team. “R: a language and environment for statistical computing.” Vienna, Austria: R Foundation for Statistical Computing. https://www.R-project.org/. (2023)
6. B. J. Callahan, P. J. McMurdie, M. J. Rosen, A. W. Han, A. J. A. Johnson, S. P. Holmes, DADA2: high-resolution sample inference from Illumina amplicon data. *Nat. Methods* 13, 581-583 (2016).
7. C. Quast, E. Pruesse, P. Yilmaz, J. Gerken, T. Schweer, P. Yarza, J. Peplies, F. O. Glöckner, The SILVA ribosomal RNA gene database project: improved data processing and web-based tools. *Nucleic Acids Res.* 41, D590-596 (2013).
8. K. Abarenkov, R. H. Nilsson, K-H. Larsson, A. F. S. Taylor, T. W. May, T. G. Frøslev, J. Pawlowska, et al. The UNITE database for molecular identification and Taxonomic Communication of Fungi and Other Eukaryotes: Sequences, Taxa and Classifications Reconsidered. *Nucleic Acids Res.* 52, D791-797 (2024).

9. N. P. Lemoine, M. L. Budny, E. Rose, J. Lucas, C. W. Marshall, Seasonal soil moisture thresholds inhibit bacterial activity and decomposition during drought in a tallgrass prairie. *Oikos* 2024, e10201 (2024).
10. C. G. Reed, M. L. Budny, J. T. du Toit, R. Helcoski, J. P. Schimel, I. P. J. Smit, T. Strydom, A. Tallian, D. I. Thompson, H. van Coller, N. P. Lemoine, D. E. Burkepile, Elephant megacarcasses increase local nutrient pools in African savanna soils and plants. *Biogeosciences* 22, 1583-1596 (2025).
11. L. Bahaa-el-din, R. Sollmann, L. T. B. Hunter, R. Slotow, D. W. Macdonald, P. Henschel, Effects of human land-use on Africa's only forest-dependent felid: The African golden cat *Caracal aurata. Biol. Conserv.* 199, 1-9 (2016).
12. R. Sollmann, A gentle introduction to camera-trap data analysis. *Afr. J. Ecol.* 56, 740-749 (2018).
13. P. J. McMurdie, S. Holmes, Phyloseq: an R package for reproducible interactive analysis and graphics of microbiome census data. *PloS One* 8, e61217 (2013).
14. D. Bates, M. Mächler, B. Bolker, S. Walker, Fitting linear mixed-effects models using lme4. *J. Stat. Softw*. 67, 1-48 (2015).

Table S1. Linear mixed effects model results for soil nutrient concentrations and soil pH at elephant carcass sites. Each row represents one model, where we report the response variable for the model, the marginal and conditional R^2 values, and the coefficients ± standard error, *p*-value, and Benjamini-Hochberg corrected *p*-value (*q*) for each parameter. For each response variable, we used the log response ratio of on vs. off carcass. Carcass site was included as a random effect to account for repeat sampling across years. Conditional R^2 values of "NA" indicate that carcass site did not contribute significantly to the variance. The number of observations for each model was N = 64. Asterisks indicate parameter significance (*p* or *q* < 0.001***, *p* or *q* < 0.01**, *p* or *q* < 0.05*).

**Response Variable**	**Marg. R^2**	**Cond. R^2**	**Intercept (Effect of Carcass)**	**Log (Carcass Age)**	**Soil Type**	**Log (Carcass Age) × Soil Type**
Ammonium (mg/kg)	0.22	0.46	3.29 ± 0.43<br>*p* < 0.001***<br>*q* = 0.004**	-0.96 ± 0.32<br>*p* = 0.004**<br>*q* = 0.012*	-0.30 ± 0.73<br>*p* = 0.677<br>*q* = 0.760	-0.41 ± 0.57<br>*p* = 0.469<br>*q* = 0.625
Nitrate (mg/kg)	0.15	0.62	2.15 ± 0.31<br>*p* < 0.001***<br>*q* = 0.004**	-0.43 ± 0.22<br>*p* = 0.059<br>*q* = 0.122	0.08 ± 0.54<br>*p* = 0.887<br>*q* = 0.930	1.28 ± 0.37<br>*p* = 0.001**<br>*q* = 0.004**
$\delta^{15}N$	0.26	0.89	0.62 ± 0.07<br>*p* < 0.001***<br>*q* = 0.004**	-0.11 ± 0.04<br>*p* = 0.022*<br>*q* = 0.052	0.22 ± 0.12<br>*p* = 0.065<br>*q* = 0.130	-0.11 ± 0.06<br>*p* = 0.069<br>*q* = 0.134
P-Bray 1 (mg/kg)	0.42	NA	1.06 ± 0.24<br>*p* < 0.001***<br>*q* = 0.004**	-0.02 ± 0.19<br>*p* = 0.932<br>*q* = 0.945	2.51 ± 0.40<br>*p* < 0.001***<br>*q* = 0.004**	-0.59 ± 0.35<br>*p* = 0.094<br>*q* = 0.177
pH	0.36	0.88	-0.08 ± 0.04<br>*p* = 0.034*<br>*q* = 0.078	-0.02 ± 0.02<br>*p* = 0.379<br>*q* = 0.527	0.04 ± 0.06<br>*p* = 0.504<br>*q* = 0.645	-0.19 ± 0.03<br>*p* < 0.001***<br>*q* = 0.004**

Table S2. Linear mixed effects model results for microbial and plant Shannon diversity at elephant carcass sites. Each row represents one model, where we report the response variable for the model, the marginal and conditional R^2 values, and the coefficients ± standard error, *p*-value, and Benjamini-Hochberg corrected *p*-value (*q*) for each parameter. For each response variable, we used the log response ratio of on vs. off carcass. Carcass site was included as a random effect. The number of observations for each model was N = 60 for bacterial communities, N = 55 for fungal communities, and N = 63 for plant community diversity. Sample sizes were lower for microbial communities because 4 sites from the end of the 2025 sampling season were not sent for sequencing. Five additional sites (3 from 2024 and 2 from 2025) had fungal samples dropped during rarefaction due to low sequence read counts. Herbaceous biomass had a sample size of N = 61 due to 3 misplaced datasheets from 2024. Asterisks indicate parameter significance (*p* or *q* < 0.001***, *p* or *q* < 0.01**, *p* or *q* < 0.05*).

**Community**	**Response Variable**	**Marg. R^2**	**Cond. R^2**	**Intercept (Effect of Carcass)**	**Log (Carcass Age)**	**Soil Type**	**Log (Carcass Age) × Soil Type**
Bacteria (16S)	*Shannon Diversity*	0.18	0.24	-0.06 ± 0.02<br>*p* = 0.003**<br>*q* = 0.009**	0.02 ± 0.02<br>*p* = 0.337<br>*q* = 0.526	-0.02 ± 0.03<br>*p* = 0.554<br>*q* = 0.670	0.06 ± 0.03<br>*p* = 0.055<br>*q* = 0.117
Fungi (ITS)	*Shannon Diversity*	0.26	0.84	-0.36 ± 0.06<br>*p* < 0.001***<br>*q* = 0.004**	0.18 ± 0.05<br>*p* < 0.001***<br>*q* = 0.004**	-0.06 ± 0.11<br>*p* = 0.560<br>*q* = 0.670	-0.04 ± 0.08<br>*p* = 0.583<br>*q* = 0.670
Plants	*Herbaceous Biomass*	0.33	0.83	-2.71 ± 0.38<br>*p* < 0.001***<br>*q* = 0.004**	0.98 ± 0.26<br>*p* < 0.001***<br>*q* = 0.004**	0.00 ± 0.67<br>*p* = 0.995<br>*q* = 0.995	0.56 ± 0.40<br>*p* = 0.169<br>*q* = 0.285
	Shannon Diversity	0.05	0.63	-0.52 ± 0.11<br>*p* < 0.001***<br>*q* = 0.004**	0.11 ± 0.08<br>*p* = 0.144<br>*q* = 0.263	0.07 ± 0.18<br>*p* = 0.692<br>*q* = 0.764	0.02 ± 0.13<br>*p* = 0.901<br>*q* = 0.930

Table S3. Linear mixed effects model results for microbial and plant diversity versus soil macronutrients at elephant carcass sites. Because the range of soil macronutrient values was much greater on carcass sites and encompassed the values found off carcass, data were filtered to include only samples from on carcass sites. Each row represents one community (bacteria, fungi, or plants), and each column represents one model, where we report the coefficients ± standard error, *p*-value, and Benjamini-Hochberg corrected *p*-value (*q*) plus the marginal and conditional R^2 values for each soil nutrient. Models included the log of each nutrient concentration as a fixed effect and carcass site as a random effect. Conditional R^2 values of "NA" indicate that carcass site did not contribute significantly to the variance. The number of observations was N = 60 for bacterial communities, N = 55 for fungal communities, and N = 63 for plant communities. Asterisks indicate parameter significance (*p* or *q* < 0.001***, *p* or *q* < 0.01**, *p* or *q* < 0.05*).

Community	Log (Ammonium)	Log (Nitrate)	Log (P-Bray 1)
Bacteria (16S)	-0.09 ± 0.03<br>p = 0.008**<br>q = 0.020*<br>Marg. R^2 = 0.12<br>Cond. R^2 = NA	-0.06 ± 0.07<br>p = 0.370<br>q = 0.527<br>Marg. R^2 = 0.01<br>Cond. R^2 = NA	-0.14 ± 0.10<br>p = 0.155<br>q = 0.276<br>Marg. R^2 = 0.03<br>Cond. R^2 = NA
Fungi (ITS)	-0.02 ± 0.03<br>p = 0.517<br>q = 0.649<br>Marg. R^2 = 0.01<br>Cond. R^2 = 75	-0.05 ± 0.05<br>p = 0.323<br>q = 0.517<br>Marg. R^2 = 0.01<br>Cond. R^2 = 78	-0.07 ± 0.07<br>p = 0.370<br>q = 0.527<br>Marg. R^2 = 0.01<br>Cond. R^2 = 81
Plants	-0.42 ± 0.09<br>p < 0.001***<br>q = 0.004**<br>Marg. R^2 = 0.25<br>Cond. R^2 = 0.44	-0.75 ± 0.22<br>p = 0.001**<br>q = 0.004**<br>Marg. R^2 = 0.15<br>Cond. R^2 = 0.49	-0.09 ± 0.40<br>p = 0.820<br>q = 0.875<br>Marg. R^2 = 0.00<br>Cond. R^2 = 0.40

Table S4. Linear mixed effects model results for soil respiration potential. The table shows the results of one model, where we report the response variable, the marginal and conditional R^2 values, and the coefficients ± standard error, *p*-value, and Benjamini-Hochberg corrected *p*-value (*q*) for each parameter. Carcass site was included as a random effect. The number of observations was N = 58 because 4 sites from the end of the 2025 sampling season were not analyzed for microbial respiration, and samples from 2 sites in 2024 were not analyzed due to equipment malfunction. Asterisks indicate parameter significance (*p* or *q* < 0.001***, *p* or *q* < 0.01**, *p* or *q* < 0.05*).

**Response Variable**	**Marg. R^2**	**Cond. R^2**	**Intercept (Effect of Carcass)**	**Log (Carcass Age)**	**Soil Type**	**Log (Carcass Age) × Soil Type**
Soil Respiration Potential	0.59	0.72	0.49 ± 0.15<br>*p* = 0.002**<br>*q* = 0.006**	-0.53 ± 0.13<br>*p* < 0.001***<br>*q* = 0.004**	0.06 ± 0.24<br>*p* = 0.796<br>*q* = 0.863	-0.63 ± 0.19<br>*p* = 0.002**<br>*q* = 0.006**

Table S5. Generalized linear model results for grazing intensity. The table shows the results of one model, where we report the response variable, the marginal and conditional R^2 values, and the coefficients ± standard error, *p*-value, and Benjamini-Hochberg corrected *p*-value (*q*) for each parameter. Carcass site was included as a random effect. The number of observations was N = 52 because 7 sites (all < 2 months postmortem) did not have sufficient grass on carcass to record grazing data, and an additional 5 sites had to be excluded due to error during field sampling. Asterisks indicate parameter significance (*p* or *q* < 0.001***, *p* or *q* < 0.01**, *p* or *q* < 0.05*).

**Response Variable**	**Marg. R^2**	**Cond. R^2**	**Intercept (Effect of Carcass)**	**Log (Carcass Age)**	**Soil Type**	**Log (Carcass Age) × Soil Type**
Grazing Intensity	0.22	0.45	0.95 ± 0.19<br>*p* < 0.001***<br>*q* = 0.004**	0.47 ± 0.16<br>*p* = 0.005**<br>*q* = 0.013*	-0.24 ± 0.32<br>*p* = 0.455<br>*q* = 0.620	0.20 ± 0.36<br>*p* = 0.586<br>*q* = 0.670

Table S6. Linear mixed effects model results for foliar nutrient concentrations at elephant carcass sites. Each row represents one model, where we report the response variable, the marginal and conditional R^2 values, and the coefficients ± standard error, *p*-value, and Benjamini-Hochberg corrected *p*-value (*q*) for each parameter. Carcass site and grass species were included as random effects. Conditional R^2 values of "NA" indicate that random effects did not contribute significantly to the variance. The number of observations for each model was N = 52 due to 12 sites across the two sampling years that did not have sufficient grass material on carcass for foliar nutrient analysis. Asterisks indicate parameter significance (*p* or *q* < 0.001***, *p* or *q* < 0.01**, *p* or *q* < 0.05*).

Response Variable	Marg. R^2	Cond. R^2	Intercept (Effect of Carcass)	Log (Carcass Age)	Soil Type	Log (Carcass Age) × Soil Type
Nitrogen (%)	0.24	NA	0.49 ± 0.06<br>$p < 0.001$***<br>$q = 0.004$**	-0.09 ± 0.06<br>$p = 0.167$<br>$q = 0.285$	-0.27 ± 0.09<br>$p = 0.005$**<br>$q = 0.013$*	0.07 ± 0.08<br>$p = 0.371$<br>$q = 0.527$
$\delta^{15}N$	0.09	0.32	1.51 ± 0.31<br>$p < 0.001$***<br>$q = 0.004$**	-0.09 ± 0.16<br>$p = 0.579$<br>$q = 0.670$	-0.52 ± 0.24<br>$p = 0.040$*<br>$q = 0.088$	0.21 ± 0.22<br>$p = 0.345$<br>$q = 0.526$

Table S7. Generalized linear model results testing for correlation between grazing intensity and grass foliar nitrogen. The table shows the results of one model, where we report the response variable, the marginal and conditional R^2 values, and the coefficients ± standard error, *p*-value, and Benjamini-Hochberg corrected *p*-value (*q*) for each parameter. Carcass site was included as a random effect. The number of observations was N = 94 (47 carcasses over 2 years) because 5 sites did not have grazing data due to user sampling error, and 12 sites across the 2 sampling years did not have sufficient grass to collect grazing and/or isotope data. Asterisks indicate parameter significance (p or $q < 0.001$***, p or $q < 0.01$**, p or $q < 0.05$*).

Response Variable	Marg. R^2	Cond. R^2	Foliar Nitrogen (%)	Carcass Presence	Foliar Nitrogen × Carcass Presence
Grazing Intensity	0.48	0.55	0.48 ± 0.20<br>*p* = 0.015*<br>*q* = 0.037*	0.80 ± 0.61<br>*p* = 0.193<br>*q* = 0.317	0.18 ± 0.25<br>*p* = 0.486<br>*q* = 0.635

Table S8. Elephant carcass site metadata. Each row represents an individual carcass, for which we provide a unique identifier ("Carcass ID"), the section of the park in which the carcass was located ("KNP Section"), the soil type where the carcass was located ("Soil Type"), the date that the elephant died ("Date of Death"), and the years during which we performed soil and vegetation sampling for that carcass ("Years Sampled"). The rows are listed in order of date of death, from the oldest carcass to the freshest carcass.

Carcass ID	KNP Section	Soil Type	Date of Death	Years Sampled
2017_64	Shingwedzi	basaltic	29-Jun-17	2024; 2025
2018_42	Shingwedzi	basaltic	20-Mar-18	2024; 2025
2020_100	Pretoriuskop	granitic	3-Dec-20	2024; 2025
2020_107	Satara	basaltic	23-Dec-20	2024; 2025
2021_14	N'wanetsi	basaltic	17-Mar-21	2025
2021_17	Satara	basaltic	27-Mar-21	2024; 2025
2021_28	Satara	basaltic	7-May-21	2024; 2025
2022_11	N'wanetsi	basaltic	15-Feb-22	2024; 2025
2022_33	Shangoni	granitic	9-Apr-22	2024
2022_30	Skukuza	granitic	15-Apr-22	2024; 2025
2022_32	Malelane	granitic	15-Apr-22	2024; 2025
2022_37	Letaba	basaltic	28-Apr-22	2024; 2025
2022_54	N'wanetsi	basaltic	30-Jun-22	2024; 2025
2022_61	Shingwedzi	basaltic	7-Jul-22	2024; 2025
2022_75	Shangoni	granitic	6-Aug-22	2024
2023_5	Letaba	basaltic	10-Jan-23	2024; 2025
2023_15	Pretoriuskop	granitic	25-Jan-23	2024; 2025
2023_19	Shingwedzi	basaltic	21-Feb-23	2024; 2025
2023_21	Skukuza	granitic	21-Feb-23	2024; 2025
2023_32	Shingwedzi	basaltic	19-Apr-23	2024; 2025
2023_38	Shingwedzi	basaltic	26-May-23	2024; 2025
2023_66	Letaba	basaltic	16-Jul-23	2024
2023_131	Shingwedzi	basaltic	10-Nov-23	2025
2023_137	Shingwedzi	basaltic	6-Dec-23	2024; 2025
2023_141	Malelane	granitic	18-Dec-23	2024; 2025
2024_3	Stolsnek	granitic	31-Jan-24	2024; 2025
2024_4	Stolsnek	granitic	31-Jan-24	2024; 2025

2024_5	Stolsnek	granitic	31-Jan-24	2024; 2025
2024_6	Stolsnek	granitic	31-Jan-24	2024; 2025
2024_8	Shingwedzi	basaltic	19-Mar-24	2025
2024_98	Tshokwane	basaltic	14-Apr-24	2025
2024_22	Tshokwane	basaltic	11-Aug-24	2025
2024_152	Letaba	basaltic	1-Sep-24	2025
2024_206	Tshokwane	granitic	2-Oct-24	2025
2024_205	Lower Sabie	basaltic	19-Oct-24	2025
2024_232	Satara	basaltic	7-Nov-24	2025
2024_253	Tshokwane	basaltic	9-Dec-24	2025
2025_1	N’wanetsi	basaltic	12-Feb-25	2025
2025_3	Tshokwane	basaltic	19-Feb-25	2025
2025_4	Lower Sabie	basaltic	27-Feb-25	2025

Table S9. Schedule of camera trap footage showing the dates and total number of days from which we obtained paired carcass and background footage for each elephant carcass site. Rows are listed in chronological order based on the date that the camera trap was set.

Carcass ID	Camera Start Date	Camera End Date	Days Set
2022_61	4-Aug-23	9-Oct-23	66
2023_21	24-Aug-23	11-Mar-24	200
2022_33	10-Dec-23	20-Mar-24	101
2022_32	13-Mar-24	23-Oct-24	224
2022_30	14-Mar-24	4-Oct-24	204
2024_6	2-Apr-24	11-May-24	39
2024_5	9-Oct-24	10-Dec-24	62
2021_28	1-Nov-24	16-Mar-25	135

Table S10. Results of sensitivity analysis for impact of rarefaction depth on model results for bacterial Shannon diversity at elephant carcass sites. Each row represents a linear mixed effects model for Shannon diversity calculated using a progressively higher rarefaction depth. For each model, we report the rarefaction depth, the number of samples (N), the marginal and conditional R^2 values, and the coefficients ± standard error and *p*-value for each parameter ($p < 0.001$***, $p < 0.01$**, $p < 0.05$*).

Rarefaction Depth	N	Marg. R^2	Cond. R^2	Intercept (Effect of Carcass)	Log (Carcass Age)	Soil Type	Log (Carcass Age) × Soil Type
2,003	120	0.18	0.24	-0.07 ± 0.02<br>*p* = 0.003**	0.02 ± 0.02<br>*p* = 0.337	-0.02 ± 0.03<br>*p* = 0.554	0.06 ± 0.03<br>*p* = 0.055
2,215	119	0.21	0.26	-0.06 ± 0.02<br>*p* = 0.002**	0.02 ± 0.02<br>*p* = 0.320	-0.04 ± 0.03<br>*p* = 0.249	0.06 ± 0.03<br>*p* = 0.038*
2,973	118	0.28	0.37	-0.07 ± 0.02<br>*p* = 0.001**	0.02 ± 0.02<br>*p* = 0.232	-0.01 ± 0.03<br>*p* = 0.653	0.07 ± 0.03<br>*p* = 0.009**
3,353	117	0.28	0.38	-0.06 ± 0.02<br>*p* = 0.001**	0.02 ± 0.02<br>*p* = 0.272	-0.02 ± 0.03<br>*p* = 0.561	0.07 ± 0.03<br>*p* = 0.008**
3,429	116	0.28	0.35	-0.06 ± 0.02<br>*p* = 0.001**	0.02 ± 0.02<br>*p* = 0.248	-0.02 ± 0.03<br>*p* = 0.588	0.07 ± 0.03<br>*p* = 0.010*
3,509	115	0.32	0.36	-0.06 ± 0.02<br>*p* = 0.001**	0.03 ± 0.02<br>*p* = 0.072	-0.02 ± 0.03<br>*p* = 0.522	0.06 ± 0.02<br>*p* = 0.020*
3,519	114	0.28	0.30	-0.06 ± 0.02<br>*p* = 0.001**	0.03 ± 0.02<br>*p* = 0.074	-0.02 ± 0.03<br>*p* = 0.552	0.06 ± 0.03<br>*p* = 0.035*
3,542	113	0.26	0.30	-0.06 ± 0.02<br>*p* = 0.001**	0.03 ± 0.02<br>*p* = 0.085	-0.02 ± 0.03<br>*p* = 0.466	0.05 ± 0.03<br>*p* = 0.052
3,560	112	0.27	0.35	-0.06 ± 0.02<br>*p* = 0.002**	0.03 ± 0.02<br>*p* = 0.100	-0.02 ± 0.03<br>*p* = 0.519	0.06 ± 0.03<br>*p* = 0.032*
3,627	111	0.27	0.34	-0.06 ± 0.02<br>*p* = 0.001**	0.03 ± 0.02<br>*p* = 0.089	-0.02 ± 0.03<br>*p* = 0.601	0.06 ± 0.03<br>*p* = 0.040*
3,819	110	0.28	0.36	-0.06 ± 0.02<br>*p* = 0.002**	0.03 ± 0.02<br>*p* = 0.091	-0.02 ± 0.03<br>*p* = 0.538	0.06 ± 0.03<br>*p* = 0.033*
3,995	109	0.26	0.29	-0.06 ± 0.02<br>*p* = 0.001**	0.03 ± 0.02<br>*p* = 0.102	-0.01 ± 0.03<br>*p* = 0.652	0.06 ± 0.03<br>*p* = 0.040*
4,303	108	0.26	0.26	-0.06 ± 0.02<br>*p* = 0.001**	0.03 ± 0.02<br>*p* = 0.113	-0.02 ± 0.03<br>*p* = 0.623	0.06 ± 0.03<br>*p* = 0.040*
4,307	107	0.26	0.31	-0.06 ± 0.02<br>*p* = 0.001**	0.02 ± 0.02<br>*p* = 0.121	-0.01 ± 0.03<br>*p* = 0.744	0.06 ± 0.03<br>*p* = 0.034*
4,406	106	0.26	0.37	-0.06 ± 0.02<br>*p* = 0.001**	0.02 ± 0.02<br>*p* = 0.135	-0.01 ± 0.03<br>*p* = 0.761	0.06 ± 0.03<br>*p* = 0.034*

4,416	105	0.17	0.32	-0.07 ± 0.02 $p = 0.001$**	0.03 ± 0.02 $p = 0.114$	0.00 ± 0.03 $p = 0.963$	0.04 ± 0.03 $p = 0.163$
4,527	104	0.18	0.32	-0.07 ± 0.02 $p = 0.001$**	0.03 ± 0.02 $p = 0.088$	0.00 ± 0.03 $p = 0.940$	0.04 ± 0.03 $p = 0.184$
4,529	103	0.17	0.27	-0.07 ± 0.02 $p = 0.001$**	0.03 ± 0.02 $p = 0.113$	0.00 ± 0.03 $p = 0.897$	0.04 ± 0.03 $p = 0.171$
4,679	102	0.17	0.26	-0.07 ± 0.02 $p = 0.001$**	0.03 ± 0.02 $p = 0.110$	0.00 ± 0.03 $p = 0.932$	0.04 ± 0.03 $p = 0.163$
4,830	101	0.17	0.26	-0.07 ± 0.02 $p = 0.001$**	0.03 ± 0.02 $p = 0.116$	0.00 ± 0.03 $p = 0.912$	0.04 ± 0.03 $p = 0.181$
5,149	100	0.17	NA	-0.07 ± 0.02 $p = 0.001$**	0.03 ± 0.02 $p = 0.117$	0.00 ± 0.03 $p = 0.956$	0.04 ± 0.03 $p = 0.176$
5,204	99	0.18	NA	-0.07 ± 0.02 $p = 0.001$**	0.03 ± 0.02 $p = 0.114$	0.00 ± 0.03 $p = 0.965$	0.04 ± 0.03 $p = 0.165$
5,511	98	0.17	NA	-0.07 ± 0.02 $p = 0.002$**	0.02 ± 0.02 $p = 0.144$	0.00 ± 0.03 $p = 0.937$	0.04 ± 0.03 $p = 0.168$
5,663	97	0.14	NA	-0.07 ± 0.02 $p = 0.002$**	0.03 ± 0.02 $p = 0.116$	0.01 ± 0.04 $p = 0.877$	0.04 ± 0.04 $p = 0.283$
5,668	96	0.14	NA	-0.07 ± 0.02 $p = 0.001$**	0.03 ± 0.02 $p = 0.127$	0.01 ± 0.04 $p = 0.759$	0.04 ± 0.03 $p = 0.275$
6,124	95	0.16	NA	-0.08 ± 0.02 $p = 0.001$**	0.03 ± 0.02 $p = 0.074$	-0.02 ± 0.04 $p = 0.654$	0.03 ± 0.03 $p = 0.343$
6,193	94	0.16	NA	-0.07 ± 0.02 $p = 0.001$**	0.03 ± 0.02 $p = 0.087$	0.01 ± 0.04 $p = 0.718$	0.04 ± 0.04 $p = 0.324$
6,293	93	0.16	NA	-0.07 ± 0.02 $p = 0.001$**	0.03 ± 0.02 $p = 0.081$	-0.01 ± 0.04 $p = 0.695$	0.03 ± 0.04 $p = 0.342$
6,392	92	0.21	NA	-0.09 ± 0.02 $p < 0.001$***	0.04 ± 0.02 $p = 0.028$*	0.03 ± 0.04 $p = 0.446$	0.03 ± 0.03 $p = 0.458$
6,534	91	0.28	0.61	-0.08 ± 0.02 $p < 0.001$***	0.04 ± 0.02 $p = 0.022$*	0.00 ± 0.04 $p = 0.975$	0.06 ± 0.04 $p = 0.119$
6,552	90	0.27	0.62	-0.09 ± 0.02 $p < 0.001$***	0.04 ± 0.02 $p = 0.024$*	0.00 ± 0.04 $p = 0.993$	0.06 ± 0.04 $p = 0.129$
6,642	89	0.28	0.59	-0.09 ± 0.02 $p < 0.001$***	0.04 ± 0.02 $p = 0.023$*	0.00 ± 0.04 $p = 0.988$	0.06 ± 0.04 $p = 0.126$
6,890	88	0.27	0.59	-0.09 ± 0.02 $p < 0.001$***	0.04 ± 0.02 $p = 0.024$*	0.00 ± 0.04 $p = 0.964$	0.06 ± 0.04 $p = 0.121$
7,067	87	0.27	0.54	-0.08 ± 0.02 $p < 0.001$***	0.04 ± 0.02 $p = 0.025$*	0.00 ± 0.04 $p = 0.993$	0.06 ± 0.04 $p = 0.145$
7,139	86	0.27	0.53	-0.08 ± 0.02 $p < 0.001$***	0.04 ± 0.02 $p = 0.025$*	0.00 ± 0.04 $p = 0.993$	0.06 ± 0.04 $p = 0.153$
7,187	85	0.27	0.48	-0.08 ± 0.02 $p < 0.001$***	0.04 ± 0.02 $p = 0.023$*	0.00 ± 0.04 $p = 0.954$	0.06 ± 0.04 $p = 0.161$
7,301	84	0.27	0.51	-0.08 ± 0.02 $p = 0.001$**	0.04 ± 0.02 $p = 0.025$*	0.00 ± 0.04 $p = 0.977$	0.06 ± 0.04 $p = 0.146$

7,335	83	0.27	0.48	-0.08 ± 0.02 *p* = 0.001**	0.04 ± 0.02 *p* = 0.026*	0.00 ± 0.04 *p* = 0.999	0.06 ± 0.04 *p* = 0.153
7,364	82	0.28	0.52	-0.08 ± 0.02 *p* = 0.001**	0.04 ± 0.02 *p* = 0.023*	0.01 ± 0.04 *p* = 0.789	0.06 ± 0.04 *p* = 0.154
7,415	81	0.28	0.49	-0.08 ± 0.02 *p* = 0.001**	0.04 ± 0.02 *p* = 0.025*	0.01 ± 0.04 *p* = 0.781	0.06 ± 0.04 *p* = 0.151
7,779	80	0.29	0.52	-0.08 ± 0.02 *p* = 0.001**	0.04 ± 0.02 *p* = 0.022*	-0.01 ± 0.04 *p* = 0.771	0.06 ± 0.04 *p* = 0.162
8,017	79	0.28	0.51	-0.09 ± 0.02 *p* = 0.001**	0.04 ± 0.02 *p* = 0.031*	0.02 ± 0.04 *p* = 0.722	0.06 ± 0.04 *p* = 0.153
8,107	78	0.33	0.89	-0.08 ± 0.02 *p* = 0.001**	0.04 ± 0.02 *p* = 0.019*	0.01 ± 0.04 *p* = 0.906	0.07 ± 0.03 *p* = 0.037*
8,365	77	0.33	0.88	-0.08 ± 0.02 *p* = 0.001**	0.04 ± 0.02 *p* = 0.019*	0.00 ± 0.04 *p* = 0.966	0.07 ± 0.03 *p* = 0.032*
8,439	76	0.32	0.87	-0.08 ± 0.02 *p* = 0.001**	0.04 ± 0.02 *p* = 0.020*	0.00 ± 0.04 *p* = 0.916	0.07 ± 0.03 *p* = 0.045*
8,549	75	0.32	0.88	-0.08 ± 0.02 *p* = 0.001**	0.04 ± 0.02 *p* = 0.019*	0.00 ± 0.04 *p* = 0.956	0.07 ± 0.03 *p* = 0.040*
8,558	74	0.33	0.91	-0.08 ± 0.02 *p* = 0.002**	0.04 ± 0.02 *p* = 0.023*	-0.01 ± 0.04 *p* = 0.861	0.07 ± 0.03 *p* = 0.027*
8,762	73	0.32	0.89	-0.08 ± 0.02 *p* = 0.001**	0.04 ± 0.02 *p* = 0.020*	0.00 ± 0.04 *p* = 0.923	0.07 ± 0.03 *p* = 0.049*
8,935	72	0.33	0.89	-0.08 ± 0.02 *p* = 0.001**	0.05 ± 0.02 *p* = 0.020*	-0.01 ± 0.04 *p* = 0.896	0.07 ± 0.03 *p* = 0.044*
9,041	71	0.32	0.90	-0.08 ± 0.02 *p* = 0.002**	0.04 ± 0.02 *p* = 0.025*	-0.01 ± 0.05 *p* = 0.921	0.07 ± 0.03 *p* = 0.045*
9,120	70	0.32	0.89	-0.08 ± 0.02 *p* = 0.004**	0.04 ± 0.02 *p* = 0.027*	-0.01 ± 0.05 *p* = 0.871	0.07 ± 0.03 *p* = 0.046*
9,175	69	0.32	0.91	-0.08 ± 0.02 *p* = 0.003**	0.05 ± 0.02 *p* = 0.025*	-0.01 ± 0.05 *p* = 0.878	0.07 ± 0.03 *p* = 0.045*
9,200	68	0.31	0.90	-0.08 ± 0.03 *p* = 0.004**	0.04 ± 0.02 *p* = 0.030*	-0.01 ± 0.05 *p* = 0.864	0.07 ± 0.03 *p* = 0.050
9,253	67	0.34	1	-0.08 ± 0.03 *p* = 0.004**	0.05 ± 0.02 *p* = 0.027*	-0.01 ± 0.05 *p* = 0.829	0.08 ± 0.02 *p* = 0.001**
9,423	66	0.39	1	-0.09 ± 0.02 *p* = 0.001**	0.05 ± 0.02 *p* = 0.015*	0.00 ± 0.05 *p* = 0.984	0.08 ± 0.02 *p* = 0.001**
9,512	65	0.40	1	-0.09 ± 0.03 *p* = 0.002**	0.05 ± 0.02 *p* = 0.012*	-0.01 ± 0.05 *p* = 0.918	0.07 ± 0.02 *p* = 0.002**
9,776	64	0.39	1	-0.09 ± 0.03 *p* = 0.003**	0.05 ± 0.02 *p* = 0.014*	-0.01 ± 0.05 *p* = 0.886	0.07 ± 0.02 *p* = 0.002**
9,855	63	0.39	1	-0.09 ± 0.03 *p* = 0.003**	0.05 ± 0.02 *p* = 0.016*	-0.01 ± 0.06 *p* = 0.896	0.07 ± 0.02 *p* = 0.002**
9,914	62	0.39	1	-0.09 ± 0.03 *p* = 0.003**	0.05 ± 0.02 *p* = 0.014*	-0.01 ± 0.06 *p* = 0.899	0.07 ± 0.02 *p* = 0.003**

10,099	61	0.40	1	-0.09 ± 0.03<br>p = 0.003**	0.05 ± 0.02<br>p = 0.015*	-0.01 ± 0.05<br>p = 0.862	0.07 ± 0.02<br>p = 0.002**
10,266	60	0.40	1	-0.09 ± 0.03<br>p = 0.003**	0.05 ± 0.02<br>p = 0.014*	-0.01 ± 0.05<br>p = 0.907	0.07 ± 0.02<br>p = 0.002**

Table S11. Results of sensitivity analysis for impact of rarefaction depth on model results for fungal Shannon diversity at elephant carcass sites. Each row represents a linear mixed effects model for Shannon diversity calculated using a progressively higher rarefaction depth. For each model, we report the rarefaction depth, the number of samples (N), the marginal and conditional R^2 values, and the coefficients ± standard error and *p*-value for each parameter ($p < 0.001$***, $p < 0.01$**, $p < 0.05$*).

Rarefaction Depth	N	Marg. R^2	Cond. R^2	Intercept (Effect of Carcass)	Log (Carcass Age)	Soil Type	Log (Carcass Age) × Soil Type
1,129	115	0.26	0.84	-0.36 ± 0.06<br>*p* < 0.001***	0.18 ± 0.05<br>*p* = 0.001**	-0.06 ± 0.11<br>*p* = 0.560	-0.04 ± 0.08<br>*p* = 0.583
1,342	114	0.24	0.88	-0.36 ± 0.07<br>*p* < 0.001***	0.18 ± 0.05<br>*p* = 0.001**	-0.07 ± 0.12<br>*p* = 0.549	-0.03 ± 0.08<br>*p* = 0.660
1,894	113	0.25	0.86	-0.35 ± 0.07<br>*p* < 0.001***	0.18 ± 0.05<br>*p* = 0.001**	-0.08 ± 0.12<br>*p* = 0.470	-0.02 ± 0.08<br>*p* = 0.786
1,984	112	0.25	0.86	-0.35 ± 0.07<br>*p* < 0.001***	0.18 ± 0.05<br>*p* = 0.001**	-0.08 ± 0.11<br>*p* = 0.501	-0.03 ± 0.08<br>*p* = 0.686
2,162	111	0.25	0.88	-0.35 ± 0.07<br>*p* < 0.001***	0.18 ± 0.05<br>*p* = 0.001**	-0.08 ± 0.12<br>*p* = 0.491	-0.04 ± 0.08<br>*p* = 0.611
2,245	110	0.22	0.90	-0.35 ± 0.07<br>*p* < 0.001***	0.17 ± 0.05<br>*p* = 0.002**	-0.09 ± 0.13<br>*p* = 0.462	-0.04 ± 0.08<br>*p* = 0.653
2,258	109	0.24	0.88	-0.35 ± 0.07<br>*p* < 0.001***	0.18 ± 0.06<br>*p* = 0.002**	-0.09 ± 0.12<br>*p* = 0.456	-0.05 ± 0.08<br>*p* = 0.544
2,274	108	0.25	0.89	-0.35 ± 0.07<br>*p* < 0.001***	0.18 ± 0.06<br>*p* = 0.002**	-0.09 ± 0.12<br>*p* = 0.476	-0.04 ± 0.08<br>*p* = 0.599
2,364	107	0.25	0.88	-0.36 ± 0.07<br>*p* < 0.001***	0.19 ± 0.06<br>*p* = 0.002**	-0.07 ± 0.13<br>*p* = 0.556	-0.05 ± 0.08<br>*p* = 0.540
2,566	106	0.24	0.87	-0.36 ± 0.07<br>*p* < 0.001***	0.18 ± 0.06<br>*p* = 0.002**	-0.08 ± 0.12<br>*p* = 0.523	-0.04 ± 0.08<br>*p* = 0.605
2,717	105	0.19	0.92	-0.36 ± 0.07<br>*p* < 0.001***	0.17 ± 0.05<br>*p* = 0.003**	-0.04 ± 0.13<br>*p* = 0.750	-0.12 ± 0.08<br>*p* = 0.141
3,075	104	0.18	0.92	-0.36 ± 0.07<br>*p* < 0.001***	0.17 ± 0.05<br>*p* = 0.004**	-0.04 ± 0.13<br>*p* = 0.755	-0.12 ± 0.08<br>*p* = 0.155
3,182	103	0.18	0.91	-0.36 ± 0.07<br>*p* < 0.001***	0.17 ± 0.06<br>*p* = 0.004**	-0.04 ± 0.13<br>*p* = 0.786	-0.13 ± 0.08<br>*p* = 0.142
3,188	102	0.17	0.91	-0.36 ± 0.08<br>*p* < 0.001***	0.17 ± 0.06<br>*p* = 0.005**	-0.03 ± 0.13<br>*p* = 0.807	-0.13 ± 0.09<br>*p* = 0.139
3,230	101	0.17	0.91	-0.36 ± 0.08<br>*p* < 0.001***	0.17 ± 0.06<br>*p* = 0.005**	-0.04 ± 0.13<br>*p* = 0.794	-0.12 ± 0.09<br>*p* = 0.161

3,303	100	0.17	0.91	-0.35 ± 0.08 $p < 0.001$***	0.17 ± 0.06 $p = 0.006$**	-0.04 ± 0.13 $p = 0.747$	-0.12 ± 0.09 $p = 0.172$
3,322	99	0.17	0.91	-0.35 ± 0.08 $p < 0.001$***	0.17 ± 0.06 $p = 0.006$**	-0.04 ± 0.14 $p = 0.760$	-0.12 ± 0.09 $p = 0.174$
3,370	98	0.16	0.91	-0.35 ± 0.08 $p < 0.001$***	0.17 ± 0.06 $p = 0.007$**	-0.04 ± 0.14 $p = 0.779$	-0.13 ± 0.09 $p = 0.155$
3,437	97	0.20	0.93	-0.35 ± 0.08 $p < 0.001$***	0.16 ± 0.06 $p = 0.008$**	-0.18 ± 0.16 $p = 0.264$	0.17 ± 0.19 $p = 0.359$
3,457	96	0.21	0.93	-0.36 ± 0.08 $p < 0.001$***	0.17 ± 0.06 $p = 0.005$**	-0.17 ± 0.16 $p = 0.284$	0.16 ± 0.18 $p = 0.385$
3,500	95	0.25	0.92	-0.31 ± 0.08 $p < 0.001$***	0.16 ± 0.05 $p = 0.005$**	-0.22 ± 0.14 $p = 0.130$	0.18 ± 0.17 $p = 0.300$
3,501	94	0.23	0.92	-0.31 ± 0.08 $p < 0.001$***	0.16 ± 0.05 $p = 0.005$**	-0.19 ± 0.14 $p = 0.220$	0.08 ± 0.20 $p = 0.686$
3,620	93	0.23	0.92	-0.31 ± 0.08 $p < 0.001$***	0.16 ± 0.05 $p = 0.004$**	-0.17 ± 0.15 $p = 0.254$	0.07 ± 0.20 $p = 0.745$
3,627	92	0.23	0.91	-0.31 ± 0.08 $p < 0.001$***	0.16 ± 0.05 $p = 0.006$**	-0.18 ± 0.15 $p = 0.238$	0.07 ± 0.20 $p = 0.741$
3,663	91	0.24	0.90	-0.31 ± 0.08 $p < 0.001$***	0.16 ± 0.06 $p = 0.007$**	-0.18 ± 0.15 $p = 0.245$	0.07 ± 0.21 $p = 0.751$
3,747	90	0.24	0.90	-0.31 ± 0.08 $p = 0.001$**	0.16 ± 0.06 $p = 0.006$**	-0.18 ± 0.15 $p = 0.255$	0.07 ± 0.21 $p = 0.756$
3,947	89	0.24	0.90	-0.31 ± 0.08 $p = 0.001$**	0.16 ± 0.06 $p = 0.008$**	-0.18 ± 0.15 $p = 0.250$	0.07 ± 0.21 $p = 0.751$
3,999	88	0.26	0.90	-0.30 ± 0.08 $p = 0.001$**	0.18 ± 0.06 $p = 0.005$**	-0.19 ± 0.15 $p = 0.227$	0.05 ± 0.21 $p = 0.822$
4,060	87	0.26	0.90	-0.29 ± 0.08 $p = 0.001$**	0.18 ± 0.06 $p = 0.006$**	-0.19 ± 0.16 $p = 0.224$	0.05 ± 0.21 $p = 0.814$
4,197	86	0.26	0.90	-0.30 ± 0.08 $p = 0.001$**	0.18 ± 0.06 $p = 0.006$**	-0.19 ± 0.16 $p = 0.225$	0.05 ± 0.21 $p = 0.800$
4,213	85	0.25	0.89	-0.30 ± 0.08 $p = 0.001$**	0.18 ± 0.06 $p = 0.007$**	-0.19 ± 0.16 $p = 0.238$	0.06 ± 0.22 $p = 0.791$
4,215	84	0.24	0.90	-0.29 ± 0.09 $p = 0.002$**	0.17 ± 0.06 $p = 0.010$*	-0.19 ± 0.16 $p = 0.236$	0.05 ± 0.22 $p = 0.809$
4,297	83	0.23	0.90	-0.29 ± 0.09 $p = 0.003$**	0.17 ± 0.06 $p = 0.013$*	-0.21 ± 0.17 $p = 0.244$	0.07 ± 0.22 $p = 0.765$
4,505	82	0.24	0.90	-0.30 ± 0.09 $p = 0.003$**	0.17 ± 0.06 $p = 0.011$*	-0.20 ± 0.17 $p = 0.259$	0.06 ± 0.23 $p = 0.784$
4,506	81	0.28	0.94	-0.28 ± 0.09 $p = 0.004$**	0.20 ± 0.06 $p = 0.006$**	-0.21 ± 0.17 $p = 0.229$	0.03 ± 0.21 $p = 0.884$
4,511	80	0.29	0.94	-0.29 ± 0.09 $p = 0.003$**	0.20 ± 0.06 $p = 0.005$**	-0.21 ± 0.17 $p = 0.223$	0.03 ± 0.21 $p = 0.871$
4,514	79	0.34	0.98	-0.29 ± 0.09 $p = 0.003$**	0.23 ± 0.06 $p = 0.001$**	-0.19 ± 0.16 $p = 0.224$	-0.01 ± 0.16 $p = 0.938$

4,544	78	0.34	0.98	-0.29 ± 0.09 $p = 0.003$**	0.23 ± 0.06 $p = 0.002$**	-0.19 ± 0.16 $p = 0.224$	-0.01 ± 0.16 $p = 0.938$
4,690	77	0.36	0.98	-0.29 ± 0.09 $p = 0.002$**	0.22 ± 0.06 $p = 0.001$**	-0.15 ± 0.15 $p = 0.353$	0.01 ± 0.16 $p = 0.954$
4,742	76	0.35	0.98	-0.29 ± 0.09 $p = 0.005$**	0.23 ± 0.06 $p = 0.002$**	-0.15 ± 0.16 $p = 0.352$	0.00 ± 0.17 $p = 0.989$
4,767	75	0.36	0.98	-0.29 ± 0.09 $p = 0.006$**	0.23 ± 0.07 $p = 0.002$**	-0.16 ± 0.17 $p = 0.340$	0.00 ± 0.17 $p = 0.998$
4,970	74	0.33	0.98	-0.28 ± 0.09 $p = 0.006$**	0.21 ± 0.07 $p = 0.004$**	-0.17 ± 0.16 $p = 0.320$	0.01 ± 0.15 $p = 0.939$
5,006	73	0.33	0.98	-0.28 ± 0.09 $p = 0.007$**	0.22 ± 0.07 $p = 0.004$**	-0.16 ± 0.16 $p = 0.328$	0.00 ± 0.15 $p = 0.987$
5,011	72	0.33	0.98	-0.28 ± 0.09 $p = 0.007$**	0.22 ± 0.07 $p = 0.004$**	-0.17 ± 0.16 $p = 0.322$	0.01 ± 0.16 $p = 0.943$
5,016	71	0.33	0.98	-0.28 ± 0.09 $p = 0.007$**	0.22 ± 0.07 $p = 0.004$**	-0.16 ± 0.16 $p = 0.335$	0.00 ± 0.15 $p = 0.984$
5,106	70	0.60	0.98	-0.37 ± 0.06 $p < 0.001$***	0.24 ± 0.04 $p < 0.001$***	-0.09 ± 0.11 $p = 0.417$	0.01 ± 0.13 $p = 0.922$
5,112	69	0.65	0.98	-0.37 ± 0.06 $p < 0.001$***	0.24 ± 0.04 $p < 0.001$***	-0.02 ± 0.11 $p = 0.886$	-0.03 ± 0.12 $p = 0.789$
5,159	68	0.65	0.98	-0.37 ± 0.06 $p < 0.001$***	0.24 ± 0.04 $p < 0.001$***	-0.01 ± 0.11 $p = 0.897$	-0.03 ± 0.12 $p = 0.787$
5,160	67	0.64	0.96	-0.37 ± 0.06 $p < 0.001$***	0.24 ± 0.04 $p < 0.001$***	-0.02 ± 0.11 $p = 0.890$	-0.03 ± 0.14 $p = 0.853$
5,221	66	0.65	0.97	-0.38 ± 0.06 $p < 0.001$***	0.25 ± 0.04 $p < 0.001$***	-0.01 ± 0.11 $p = 0.968$	-0.04 ± 0.13 $p = 0.771$
5,252	65	0.65	0.97	-0.38 ± 0.06 $p < 0.001$***	0.25 ± 0.04 $p < 0.001$***	0.01 ± 0.12 $p = 0.963$	-0.03 ± 0.14 $p = 0.848$
5,444	64	0.66	0.97	-0.40 ± 0.07 $p < 0.001$***	0.25 ± 0.04 $p < 0.001$***	0.03 ± 0.13 $p = 0.834$	-0.04 ± 0.14 $p = 0.810$
5,459	63	0.56	0.96	-0.39 ± 0.08 $p < 0.001$***	0.25 ± 0.06 $p = 0.001$**	0.02 ± 0.13 $p = 0.859$	-0.03 ± 0.15 $p = 0.823$
5,481	62	0.56	0.96	-0.40 ± 0.08 $p < 0.001$***	0.25 ± 0.06 $p = 0.001$**	0.03 ± 0.13 $p = 0.852$	-0.03 ± 0.15 $p = 0.819$
5,575	61	0.56	0.97	-0.40 ± 0.08 $p < 0.001$***	0.25 ± 0.06 $p = 0.001$**	0.05 ± 0.15 $p = 0.732$	-0.04 ± 0.15 $p = 0.797$
25,593	60	0.56	0.96	-0.40 ± 0.08 $p < 0.001$***	0.25 ± 0.06 $p = 0.001$**	0.05 ± 0.15 $p = 0.734$	-0.04 ± 0.15 $p = 0.779$

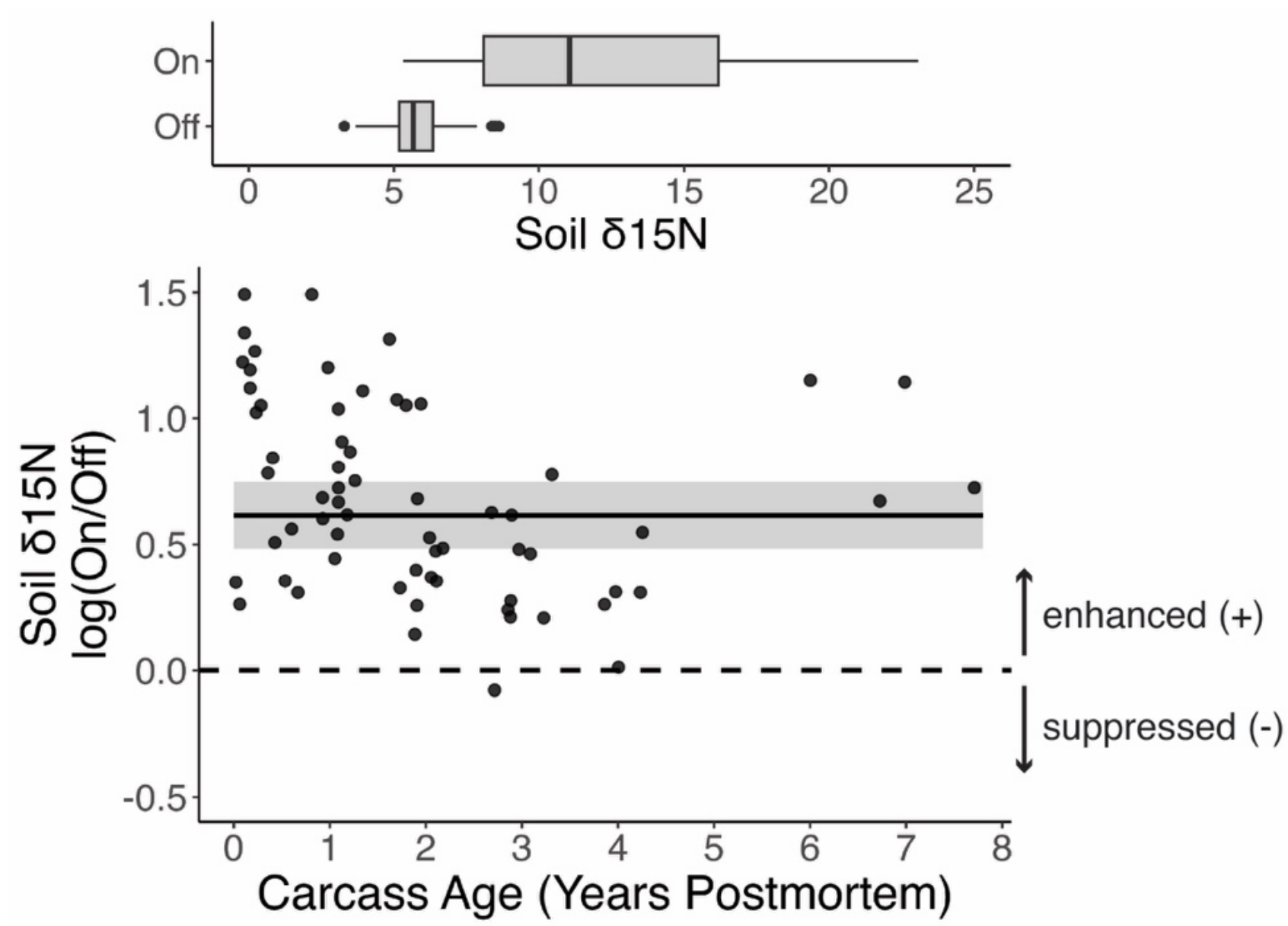

Figure S1. Elephant carcasses had a strong positive effect on soil $\delta^{15}N$ that persisted even at the oldest carcasses. Points represent on vs. off carcass log response ratios from individual carcass sites and are offset to be visible when they would otherwise overlap. Points above the dashed horizontal line at 0 indicate positive effects of carcasses on soil $\delta^{15}N$, while points below 0 indicate negative effects. Lines show predictions calculated from the linear mixed effects model, which included the log of carcass age, soil type, and a log of carcass age × soil type interaction as covariates. Only significant relationships are shown on the plots. Shading indicates the 95% confidence interval. Above the effect size plot is a panel showing the distribution of values. On the boxplot, boxes show the interquartile range, middle line indicates the median, whiskers extend up to 1.5x the interquartile range, and points are shown for outliers that fall >1.5x outside the interquartile range.

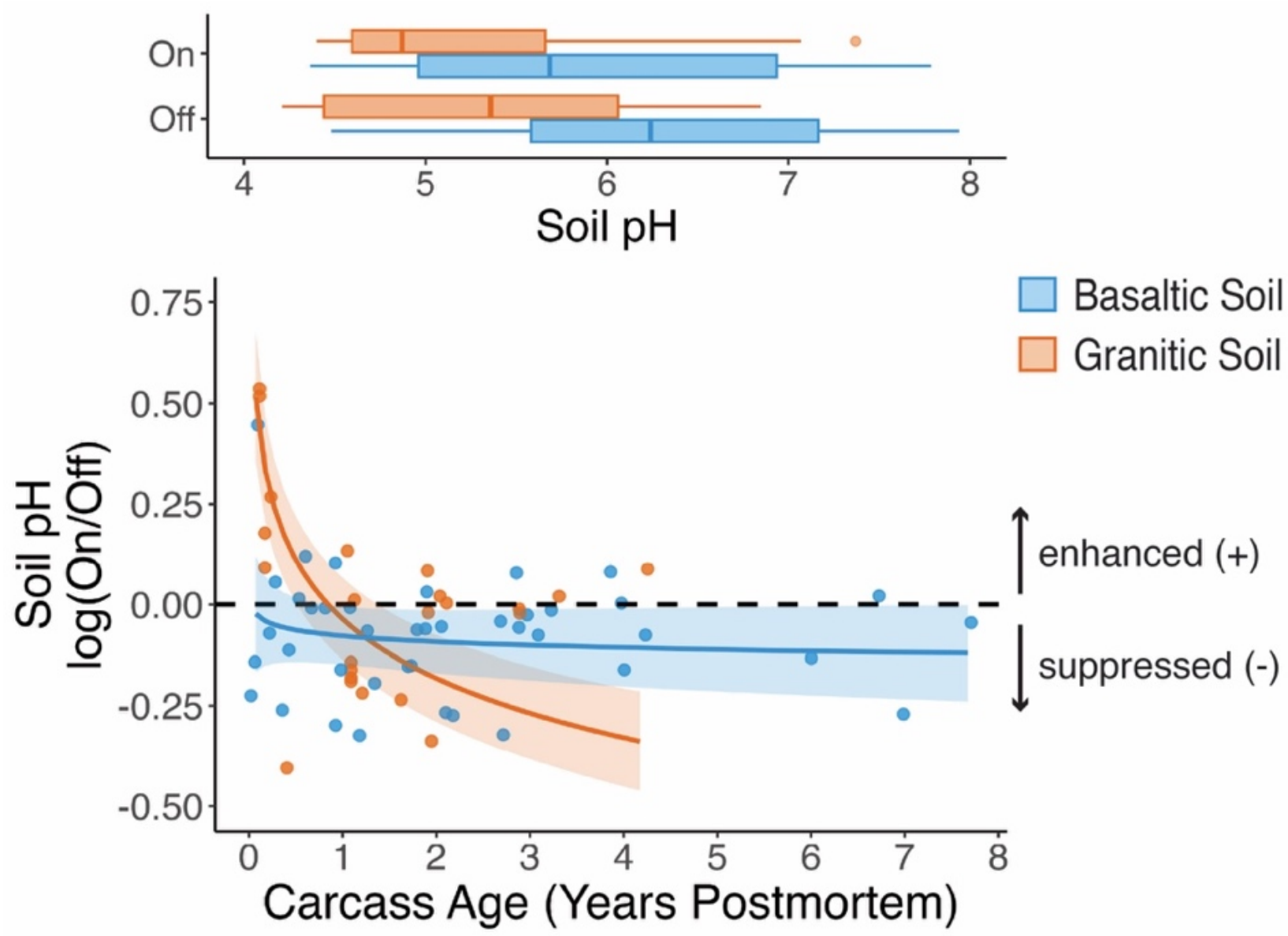

Figure S2. There was no significant effect of the carcass on soil pH, though there was a significant carcass age × soil type interaction. Points represent on vs. off carcass log response ratios from individual carcass sites and are offset to be visible when they would otherwise overlap. Points above the dashed horizontal line at 0 indicate positive effects of carcasses on soil pH, while points below 0 indicate negative effects. Lines show predictions calculated from the linear mixed effects model, which included the log of carcass age, soil type, and a log of carcass age × soil type interaction as covariates. Only significant relationships are shown on the plots. Thus, we visualize the significant carcass age × soil type interaction by plotting the soil types separately. Shading indicates the 95% confidence interval. Above the effect size plot is a panel showing the distribution of values. On the boxplot, boxes show the interquartile range, middle line indicates the median, whiskers extend up to 1.5x the interquartile range, and points are shown for outliers that fall >1.5x outside the interquartile range.

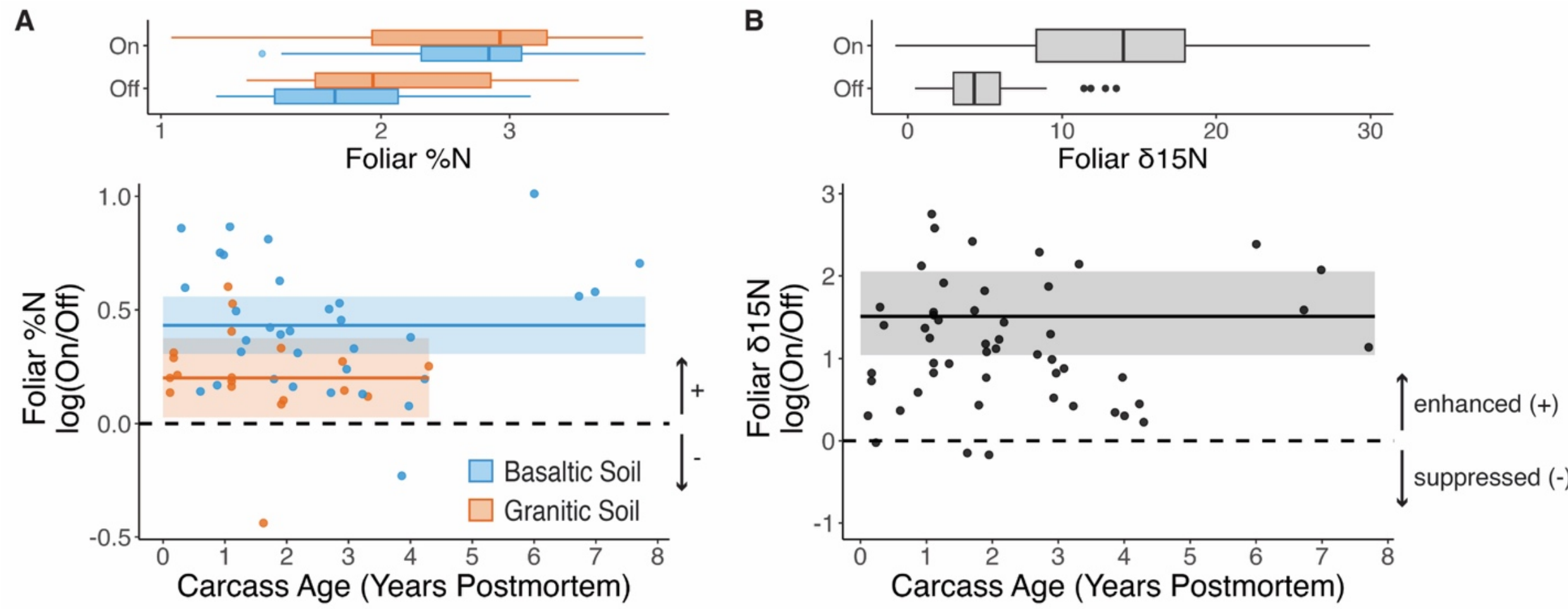

Figure S3. Impacts of elephant carcasses on foliar N. (A) Grass foliar % N and B) $\delta^{15}N$ were both strongly elevated on carcass sites and remained so even at the oldest carcass sites, and foliar % N was overall greater in grasses growing in basaltic soils. Points represent on vs. off carcass log response ratios from individual carcass sites and are offset to be visible when they would otherwise overlap. Points above the dashed horizontal line at 0 indicate positive effects of carcasses on foliar N, while points below 0 indicate negative effects. Lines show predictions calculated from the linear mixed effects model, which included the log of carcass age, soil type, and a log of carcass age × soil type interaction as covariates. Only significant relationships are shown on the plots. Shading indicates the 95% confidence interval. Above the effect size plots are panels showing the distribution of values. On the boxplots, boxes show the interquartile range, middle line indicates the median, whiskers extend up to 1.5x the interquartile range, and points are shown for outliers that fall >1.5x outside the interquartile range.

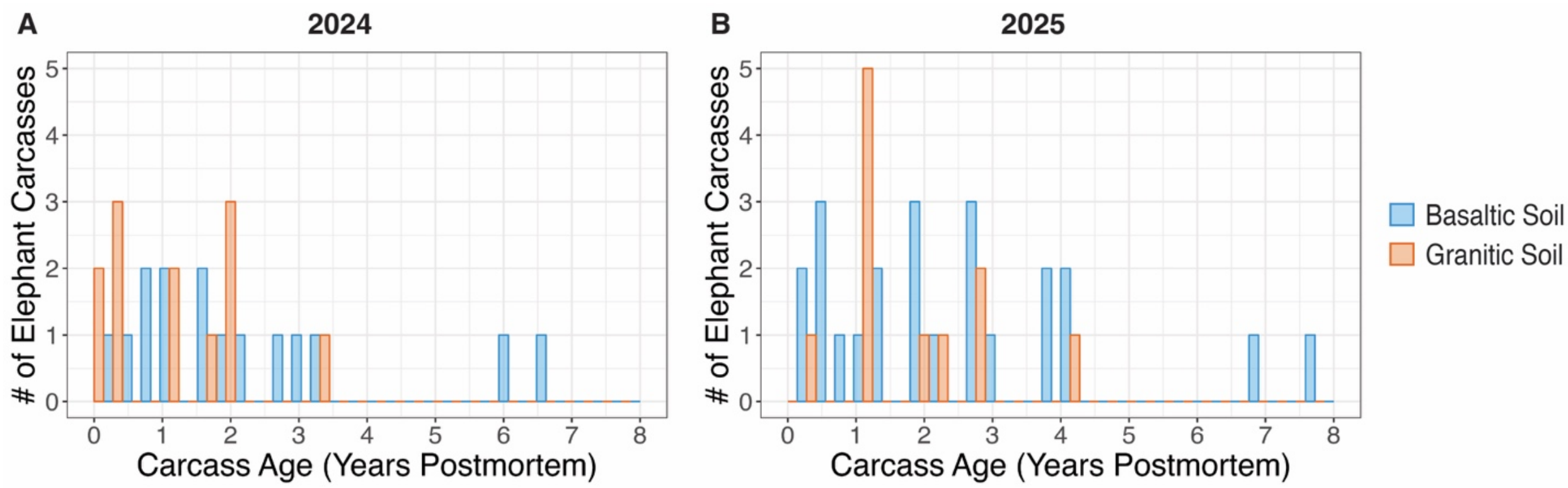

Figure S4. Histograms showing age distribution of elephant carcasses during the (A) 2024 and (B) 2025 sampling seasons. Blue bins indicate carcasses on basaltic soils, and orange bins indicate carcasses on granitic soils.

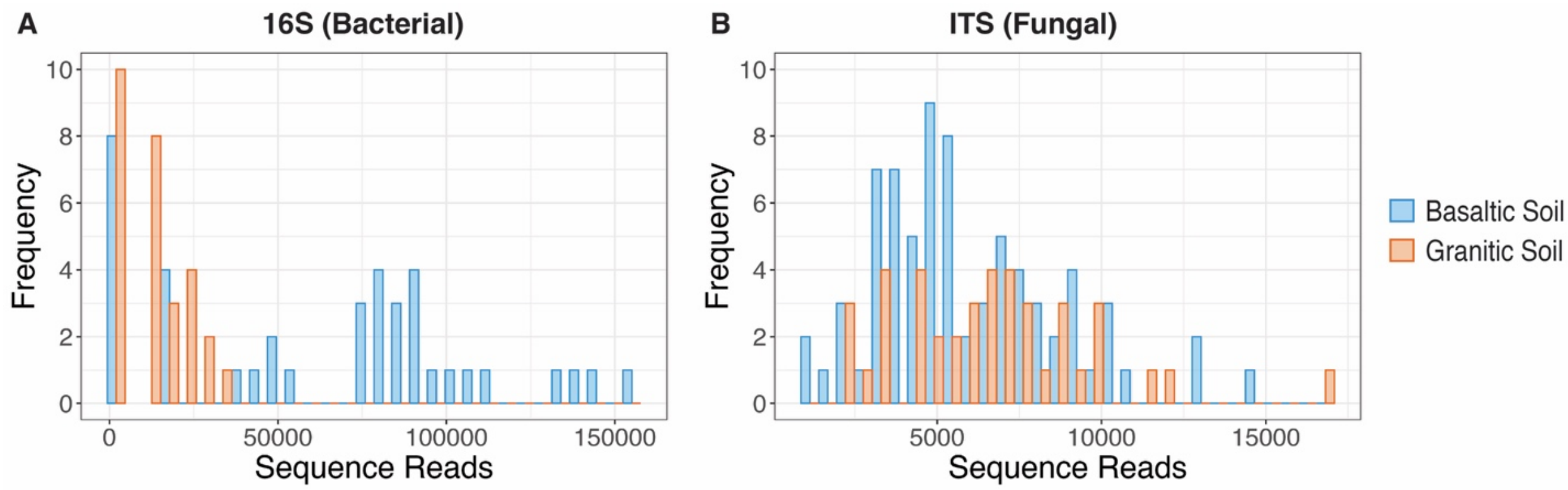

Figure S5. Histograms showing distributions of sequence read counts in soil samples for (A) bacterial and (B) fungal communities. Blue bins indicate carcasses on basaltic soils, and orange bins indicate carcasses on granitic soils.
